# Supplementary material for: Hyporesponsiveness to erythropoiesis-stimulating agent in non-dialysis-dependent CKD patients: The BRIGHTEN study
Source: PLoS One. 2022 Nov 29;17(11):e0277921. doi: 10.1371/journal.pone.0277921 (PMC9707758; doi:10.1371/journal.pone.0277921)
Supplement: S1 Appendix — (PDF) [file pone.0277921.s002.pdf]

oBservational clinical Research In chronic kidney disease patients with renal anemia:
renal proGnosis in patients with Hyporesponsive anemia To Erythropoiesis stimulating agents, darbepoetiN alfa
BRIGHTEN

Research Protocol Attachment 1

Principal investigator: Department of Nephrology, Nagoya University Graduate School of Medicine Seiichi Matsuo
Co-principal investigator: Department of Nephrology and Endocrinology, University of Tokyo Graduate School of Medicine Masaomi Nangaku

Research support institution: Translational Research Informatics Center
Research consignor: Kyowa Hakko Kirin Co., Ltd.

May 2, 2014 Ver. 1.0 (approved by the ethics review board of the principal investigator facility)

All those involved in this research follow the spirit of the "World Medical Association Declaration of Helsinki" and "Ethical Guidelines for Clinical Research" (fully revised on July 31, 2008; Ministry of Health, Labor and Welfare).

Cardiovascular disease event list and definitions
The following are treated as events (as a general rule, the onset date of an event is the date when symptoms are observed, but the onset date of an asymptomatic event is the date when an inspection that serves as the basis of diagnosis is performed).

Fatal endpoints	Non-fatal endpoints
F1: Death due to myocardial infarction<br>F2: Sudden death<br>F3: Death due to congestive heart failure<br>F4: Death due to arrhythmia<br>F5: Cerebrovascular death<br>F6: Death due to aortic dissection<br>F7: Death due to ischemia of major organs other than the heart / brain<br>F8: Other forms of cardiovascular diseases	N1: Myocardial infarction*1<br>N2: Hospitalization due to angina pectoris<br>N3: Hospitalization for ischemic heart disease requiring invasive intervention treatment<br>N4: Hospitalization for congestive heart failure with chronic organic heart disease*2 (excluding arrhythmia)<br>N5: Hospitalization for severe arrhythmia and atrial fibrillation / atrial flutter<br>N6: Hospitalization due to cerebrovascular disease<br>N7: Hospitalization due to chronic arteriosclerosis obliterans<br>N8: Hospitalization due to aortic dissection<br>N9: Hospitalization due to ischemia of major organs other than the heart / brain

***1 New-onset (asymptomatic) myocardial infarction is treated as an event even without hospitalization.**
***2 Includes old myocardial infarction, valvular disease, epicarditis, etc.**

Cardiovascular disease event: definitions

F1 Death due to myocardial infarction

Myocardial infarction is an ischemic heart disease in which blood flow in the coronary arteries that feed the myocardium is locally reduced or interrupted for a certain period of time and causes necrosis of the myocardium in the perfusion area. Specific electrocardiogram changes, increased myocardial deviation enzymes, and angiographic findings are the basis for diagnosis.

Death due to myocardial infarction is defined as that which satisfies one of the following two conditions:

(1) Hospitalization due to definitive diagnosis or suspicion of myocardial infarction and death during hospitalization

(2) Patients with a previous history of a definitive diagnosis of angina pectoris, a definitive diagnosis or suspicion of myocardial infarction, and whose cause of death cannot be thought to be other than myocardial infarction.

N1 Myocardial infarction

Myocardial infarction is an ischemic heart disease in which blood flow in the coronary arteries that feed the myocardium is locally reduced or interrupted for a certain period of time and causes necrosis of the myocardium in the perfusion area. Specific electrocardiogram changes, increased myocardial deviation enzymes, and angiographic findings are the basis for diagnosis.

Myocardial infarction is defined as one of the following conditions:

(1) Electrocardiogram findings that meet the definitive diagnostic criteria at the time of the event (classified by the Minnesota Code)

The definitive diagnostic criteria are defined as those where one of the following apply:

（a）Standard Q or QS pattern (code 1-1）

(b) Any of the T-wave findings (code 5-1 to 5-3) in addition to Q or QS pattern (code 1-2-1 to 1-2-7); apply (1) if there is intraventricular conduction disorder

(2) Cardiac ischemic chest pain and enzyme levels that meet definitive diagnostic criteria

(3) Cardiac ischemic chest pain, enzyme levels that meet the criteria for suspicion, and electrocardiogram findings that meet the criteria for suspicion.

(4) Current electrocardiogram findings meet the definitive diagnostic criteria of myocardial infarction even though the previous recording at the time of the event did not.
This corresponds to new-onset (asymptomatic) myocardial infarction and is treated as an event, even without hospitalization.

(5) Increased levels of myocardial enzymes

CK, GOT (AST), or LDH was measured at the time of the event, of which at least one type exhibited an increase of more than twice the normal upper limit (definitive diagnosis) or an increase above the normal upper limit but within two times its value (suspicion). Additionally, if troponin T was measured at the time of the event, an increase in its value was observed (definitive diagnosis).

N2 Angina pectoris

Angina pectoris is a syndrome manifested as a characteristic chest symptom (chest/heart pain) caused by transient ischemia of the myocardium. From a pathophysiological perspective, ischemia is broadly divided into organic ischemia based on atherosclerotic changes in the coronary arteries and functional ischemia based on the spasm of the coronary arteries (so-called coronary spasm angina pectoris). This study focuses on the former organic angina pectoris and defines it as those that satisfy all the following symptoms (chest pain and chest discomfort).

(1) Pain, discomfort, or zonesthesia in any part of the sternum

(2) Appearance during exertion or stress and normally lasting over 30 s (regardless of the extent of exertion or stress)

(3) Symptoms are alleviated or disappear within 10 min in most patients after rest or when exertion is alleviated.

(4) Usually improved with nitroglycerin within 2-5 minutes

However, this criterion does not apply when nitroglycerin has never been used.

N3 Ischemic heart disease requiring invasive intervention treatment

Ischemic heart disease requiring invasive intervention treatment is defined as ischemic heart disease that requires hospitalization during the observation period for diagnosis or treatment, or measures, such as percutaneous coronary intervention (PCI) or coronary artery bypass grafting (CABG). The date of hospitalization is set as the event occurrence date in this case, and the implementation of intervention treatment is not an essential condition.

F2 Sudden death

Sudden death refers to a situation in which a patient whose condition was stable to date suddenly loses consciousness and dies within 24 hours. This also includes cases of death despite the implementation of CPR during the loss of consciousness.

Sudden deaths in this research are defined as those that meet all the following criteria:

(1) Death within 24 hours of the occurrence of serious symptoms, or death where the last person to have met the patient confirmed their asymptomatic condition

(2) No findings of diseases that may lead to death other than acute or chronic coronary artery disease

(3) “Unexpected” deaths occur to a person who does not rest at home, is hospitalized, or did not enter other facilities due to disease within 24 hours of death

F3, N4 Congestive heart failure

Heart failure is hemodynamically referred to as a condition in which cardiac output cannot be maintained to meet the metabolic demands of peripheral tissues as a result of decreased cardiac function, or a condition that could only be maintained by abnormal increases in left ventricular inflow pressure. This is clinically defined as a state in which the so-called heart failure symptoms occur as a result of decreased cardiac function and limited activity abilities. This involves systemic changes in which a regulatory response is added to a decrease in cardiac function, is often accompanied by arrhythmia, and generally has a poor prognosis.

Those in this study who satisfy more than one of the following items and who are clinically diagnosed with congestive heart failure are classified under this group:

(1) Not heart failure caused by an acute event (due to chronic events, such as old myocardial infarction, valvular disease, and epicarditis).
(2) Significant dyspnea.
(3) Significant pulmonary edema is observed on frontal chest radiography.
(4) Significantly increased BNP values are measured.
(5) Strict in-out balance control is essential.

F4 Death due to arrhythmia

This research focuses on deaths from arrhythmia, such as those listed below. The existence of electrocardiogram records or confirmation using an electrocardiogram monitor is ideal.

(1) Ventricular fibrillation
(2) Persistent ventricular tachycardia
(3) Torsade de pointes (TDP)
(4) Asystole due to atrioventricular block, sick sinus syndrome, etc.)

N5 Severe arrhythmia and atrial fibrillation / atrial flutter

This study targets the following events: The existence of electrocardiogram records or confirmation using an electrocardiogram monitor is ideal.

(1) Ventricular fibrillation
(2) Persistent ventricular tachycardia
(3) Torsade de pointes (TDP)
(4) Mobitz type II atrioventricular block/complete atrioventricular block.
(5) Sick sinus syndrome.
(6) Atrial fibrillation.
(7) Atrial flutter

F5 Cerebrovascular death ・N6 Cerebrovascular disease

This research records events by dividing between the following three types.

A Cerebral infarction
B Cerebral hemorrhage
C Subarachnoid hemorrhage

All of the following conditions should be met as a basis for diagnosis.

(1) Confirmation of acute-onset, clear, and objective findings of local nerve deficiency
(2) Findings continued for more than 24 h.
(3) Neurological findings are not due to extracranial disorders.
(4) Cerebrovascular disorders could be classified as either cerebral hemorrhage, cerebral infarction, or subarachnoid hemorrhage using CT, MRI, or autopsy records within three weeks of onset
*However, subarachnoid hemorrhages include cases in which a sudden severe headache is confirmed instead of (1)–(3).

As supplementary information, cerebral infarction is defined as infarction observed in the area where CT findings were normal or predicted from clinical findings, or where a cerebral infarction is confirmed by autopsy. Patients with no symptoms, indicated by images (such as CT or MRI), will be excluded. Additionally, cerebral hemorrhage and subarachnoid hemorrhage are classified based on findings obtained by CT, MRI, or autopsy. Cases of acute cerebral infarction or brain tumor transitioning to hemorrhage will be excluded.

N7 Chronic arteriosclerosis obliterans

This refers to a condition in which ischemic symptoms appear in the affected limb due to the progression of luminal stenosis caused by arteriosclerosis in moderately thick arteries branching from the aorta, especially in the peripheral arteries that perfuse the limbs.

This research defines chronic arteriosclerosis obliterans as that where one of the following applies:

(1) Vascular intermittent claudication
(2) Unilateral disappearance or weakness of femoral artery pulsation
(3) Unilateral disappearance or weakness of the dorsalis pedis artery or posterior cervical artery pulsation

(4) Bilateral disappearance or weakness of the dorsalis pedis artery or posterior cervical artery pulsation
(5) Ankle brachial index (ABI) ＜0.8
(6) Angiography showing stenosis (stenosis >75% or collateral circulation)
(7) Lower leg gangrene or ulcers.

F6, N8 Aortic dissection (dissecting aortic aneurysm)

This is a disease in which lacunae are formed in the aortic intima and bleeding occurs in the aortic wall due to aortic pressure, which is accompanied by media dissociation. Diagnosis in this research requires symptomatology or a clear basis on CT or MRI.

F7, N9 Ischemia of major organs other than the heart / brain

Patients with death or hospitalization due to diseases caused by ischemia from organs other than the heart or brain (e.g., ischemic enteritis) are defined as events. The suitability of such patients will be determined individually by the event evaluation committee.
