# Supplementary material for: Hyporesponsiveness to erythropoiesis-stimulating agent in non-dialysis-dependent CKD patients: The BRIGHTEN study
Source: PLoS One. 2022 Nov 29;17(11):e0277921. doi: 10.1371/journal.pone.0277921 (PMC9707758; doi:10.1371/journal.pone.0277921)
Supplement: S1 File — (PDF) [file pone.0277921.s003.pdf]

oBservational clinical Research In chronic kidney disease patients with renal anemia: renal proGnosis in patients with Hyporesponsive anemia To Erythropoiesis stimulating agents, darbepoetiN alfa Research implementation plan - List of changes

【Ver. 1.0⇒Ver. 1.1】

Created June 25, 2014

Reason for change	Changes	Prior to change	After change
To improve the system for collecting / reporting adverse events	9.2 Evaluation and reporting of adverse events in this study	If a serious adverse event specified in “9.1.2 Definition of serious adverse events” is observed, then report this according to “9.4 Emergency reporting of adverse events and subsequent responses” and “Appendix 2 Reporting and response manual when serious adverse event occurs”.	The lead principal physician and study physician will list all adverse events to be evaluated in the case report form. Furthermore, if a serious adverse event specified in “9.1.2 Definition of serious adverse events” is observed, then report this according to “9.4 Emergency reporting of serious adverse events and subsequent responses” and “Appendix 2 Reporting and response manual when serious adverse event occurs”.
	9.4.2 Reporting procedure	After confirming the occurrence of serious adverse events, the lead principal physician or study physician of each implementing medical institution will promptly list them in the “adverse events” section of the case report form of the online patient registration / report system.	After confirming the occurrence of serious adverse events, the lead principal physician or study physician of each implementing medical institution will promptly report them to the principal investigator by E-mail or FAX.
To respond to new drugs	10.1.3 Observation period (from start of treatment–96 weeks later)	2* Iron administration status – Oral medicine – Drug name<br>Ferrum, Incremin, Fero-Gradumet, Slow-Fe, Tetucur, Ferromia, Fenilene, Fenelmin, Feredaim, Ferrostec, Ferotym, Ferrofiel, Foliromin	2* Iron administration status – Oral medicine – Drug name<br>Ferrum, Incremin, Fero-Gradumet, Slow-Fe, Tetucur, Ferromia, Fenilene, Fenelmin, Feredaim, Ferrostec, Ferotym, Ferrofiel, Foliromin, Riona, other (drug name)

【Ver. 1.1⇒Ver. 1.2】

Created September 2, 2014

Reason for change	Changes	Prior to change	After change
To clarify selection / exclusion criteria and eliminate the possibility of switching drugs that would be detrimental to patient	0.3 Eligibility criteria<br><br>5.1 Eligibility criteria	Selection criteria<br>1) Patients who are scheduled to start treatment with darbepoetin alfa within eight weeks after registration and who have not received ESA within 12 weeks prior to registration	Selection criteria<br>1) Patients who are planning to newly start treatment with darbepoetin alfa within eight weeks after registration<br><br>Exclusion criteria<br>2) Patients who have a history of treatment with ESA (however, those who were temporarily administered ESA over 12 weeks prior to registration are not considered to have a treatment history of ESA)
To cover all adverse events that occur in the research subjects	0.8 Observation / inspection / reporting schedule<br><br>10.3 Observation / inspection / reporting schedule	Arrow for “Events / adverse events” (from “Start” to “Discontinuation of participation / withdrawal of consent”)<br><br>Arrow for “Outcome information” (from “Start” to “Discontinuation of participation / withdrawal of consent”)	Arrow for “Events / adverse events” (from “Registration” to “Discontinuation of participation / withdrawal of consent”)<br><br>Arrow for “Outcome information” (from “Registration” to “Discontinuation of participation / withdrawal of consent”)
	9.1.1 Definition of adverse event	An adverse event refers to any unfavorable medical event (e.g., subjective and objective symptoms, abnormal laboratory test values) observed during or within 30 days after discontinuation or termination of administration of darbepoetin alfa, regardless of a causal relationship with	An adverse event refers to any unfavorable medical event (e.g., subjective and objective symptoms, abnormal laboratory test values) from the time of registration in this study which was observed during or within 30 days after discontinuation or termination of administration of

		darbepoetin alfa.	darbepoetin alfa, regardless of a causal relationship with darbepoetin alfa.
	9.2 Evaluation and reporting of adverse events in this study	Of the adverse events which occurred during or within 30 days after discontinuation or termination of administration of darbepoetin alfa, all adverse events which occurred by the end of the simultaneous outcome survey are subject to evaluation in this study.	Of the adverse events which occurred during or within 30 days after discontinuation or termination of administration of darbepoetin alfa from the time of registration in this study, all adverse events which occurred by the end of the simultaneous outcome survey are subject to evaluation in this study.
To clarify the reason why the target drug of this study was limited to darbepoetin alfa	2. Background and rationale		There are also multiple second-generation ESAs currently used in Japan in addition to darbepoetin alfa. However, there are large differences in pharmacokinetics and effects between the agents, so it was decided that this study would be limited to the darbepoetin alfa used in the TREAT trial, which has shown representative evidence internationally for low ESA response.
To clarify the range of use of stored samples	10.2 Central measurement inspection	Additionally, if items requiring inspection are added following the start of the study, then the stored samples will be used for measurement after obtaining the approval of the principal investigator-affiliated facility, the Foundation for the Promotion of Advanced Medicine, and the ethical review committee of the research consignor. The lead principal physician and study physician are to also obtain consent for sample storage and additional	Additionally, if inspection items that are thought necessary to be added in order to achieve the objectives of this study following the start of this study, then the stored samples will be used for measurement after disclosing information by an appropriate method and after obtaining the approval of the principal investigator-affiliated facility, the Foundation for the Promotion of Advanced Medicine, and the ethical review committee of the

		measurements with stored samples when consent is obtained from the patient for research participation.	research consignor. Stored samples and data will not be used for secondary purposes outside of the objectives of this study without permission from the subjects. The lead principal physician and study physician are to also obtain consent for sample storage and additional measurements with stored samples when consent is obtained from the patient for research participation.

【Ver. 1.2⇒Ver. 2.0】

Created May 14, 2015

**Reason for change**	**Changes**	**Prior to change**	**After change**
Due to changes in research organization	Cover,<br>23.1 Principal Investigator,<br>Report / response flow chart when serious adverse event occurs,<br>Report on serious adverse events,<br>Explanatory document	Principal investigator:<br>Department of Nephrology, Nagoya University Graduate School of Medicine<br>Seiichi Matsuo	Principal investigator:<br>Division of Clinical Nephrology and Rheumatology, Niigata University Graduate School of Medical and Dental Sciences Ichiei Narita
Due to changes in research organization	Cover,<br>23.2 Co-principal investigator,<br>Explanatory document	Co-principal investigator:<br>Department of Nephrology and Endocrinology, University of Tokyo Graduate School of Medicine<br>Masaomi Nangaku	Co-principal investigator:<br>Department of Nephrology and Endocrinology, University of Tokyo Graduate School of Medicine<br>Masaomi Nangaku<br>Department of Nephrology, Nagoya University Graduate

			School of Medicine<br>Shoichi Maruyama
Due to changes in research organization	23.4 Steering committee	Seiichi Matsuo Department of Nephrology, Nagoya University Graduate School of Medicine<br>Masaomi Nangaku Department of Nephrology and Endocrinology, University of Tokyo Graduate School of Medicine<br>Hideki Hirakata Division of Nephrology and Dialysis Center, Japanese Red Cross Fukuoka Hospital<br>Ichiei Narita Division of Clinical Nephrology and Rheumatology, Niigata University Graduate School of Medical and Dental Sciences<br>Takashi Wada Department of Laboratory Medicine, Institute of Medical, Pharmaceutical and Health Sciences, Faculty of Medicine, Kanazawa University<br>Terumasa Hayashi Department of Kidney Disease and Hypertension, Osaka General Medical Center<br>Shoichi Maruyama Department of Nephrology, Nagoya University Graduate School of Medicine	Ichiei Narita Division of Clinical Nephrology and Rheumatology, Niigata University Graduate School of Medical and Dental Sciences<br>Masaomi Nangaku Department of Nephrology and Endocrinology, University of Tokyo Graduate School of Medicine<br>Shoichi Maruyama Department of Nephrology, Nagoya University Graduate School of Medicine<br>Hideki Hirakata Division of Nephrology and Dialysis Center, Japanese Red Cross Fukuoka Hospital<br>Takashi Wada Department of Laboratory Medicine, Institute of Medical, Pharmaceutical and Health Sciences, Faculty of Medicine, Kanazawa University<br>Terumasa Hayashi Department of Kidney Disease and Hypertension, Osaka General Medical Center<br>Hiroshi Sato Laboratory of Clinical Pharmacology and Therapeutics, Tohoku University Graduate School of Pharmaceutical Sciences<br>Yasushi Yamasaki Department of Internal Medicine for Kidney and Collagen Disease, Kagawa Prefectural Central Hospital<br>Takao Masaki Department of Nephrology, Hiroshima University Hospital

For package insert amendments	9.3 Expected adverse events	<Adults><br>In Japanese clinical trials, side effects (including abnormal laboratory test values) were observed in 471 out of 1462 patients (32.2%). The main side effects were hypertension in 248 patients (17.0%), shunt thrombosis / obstruction in 44 patients (3.0%), headache in 28 patients (1.9%), and malaise in 20 patients (1.4%) (at the time of approval of NESP ® injection).<br><br>(1) Serious side effects<br>Cerebral infarction (0.9%)<br><br>(2) Other side effects<br>See *1	<Adults><br>In Japanese clinical trials, side effects (including abnormal laboratory test values) were observed in 472 out of 1462 patients (32.3%). The main side effects were hypertension in 248 patients (17.0%), shunt thrombosis / obstruction in 44 patients (3.0%), headache in 29 patients (2.0%), and malaise in 20 patients (1.4%) (at the time of approval of NESP ® injection).<br><br>(1) Serious side effects<br>Cerebral infarction (0.8%)<br><br>(2) Other side effects<br>See *1

*1 <Regarding changes in “9.3 Expected adverse events” – (2) Other side effects>

＜Prior to change＞

	**Frequency of side effect (%)**		
	Over 1%	0.5%–1%	Less than 0.5% or unknown frequency
Cardiovascular system	Hypertension	Arrhythmia	Hypotension during dialysis, palpitations, angina / myocardial ischemia, arteriosclerosis obliterans
Skin		Pruritus, rash	

Liver		Liver dysfunction, increased Al-P	Increased γ -GTP, increased AST (GOT), increased ALT (GPT), gallbladder polyps
Metabolism			Decreased stored iron, increased serum potassium, increased blood phosphorus, increased uric acid, decreased appetite, secondary hyperparathyroidism
Blood	Eosinophilia		Thrombocytopenia, leukocytosis, lymphopenia, leukopenia
Kidney / urinary system		Decreased renal function (e.g., increased BUN, increased creatinine)	Hematuria
Digestive system			Nausea / vomiting, abdominal pain, gastritis, duodenal inflammation
Sensory organs	Headache, malaise		Dizziness, sensory hearing loss
Eye			Vitreous hemorrhage, conjunctivitis
Other	Shunt thrombosis / obstruction, increased LDH		Residual blood in dialysis circuit, chest discomfort, shunt pain, poor hemostasis, diabetic gangrene, fever, musculoskeletal pain, hot flashes*

＜After change＞

	**Frequency of side effect (%)**		
	Over 1%	0.5%–1%	Less than 0.5% or unknown frequency
Cardiovascular system	Hypertension (16.2%)	Arrhythmia	Angina / myocardial ischemia, hypotension during dialysis, palpitations, arteriosclerosis obliterans
Skin		Pruritus, rash	
Liver	Liver dysfunction (increased Al-P, increased γ-GTP, increased AST (GOT), increased ALT (GPT), increased bilirubin)		Gallbladder polyps
Metabolism			Increased serum potassium, increased uric acid, decreased stored iron, increased blood phosphorus, decreased appetite, secondary hyperparathyroidism
Blood		Eosinophilia, thrombocytopenia	Lymphopenia, leukopenia, leukocytosis
Kidney / urinary system		Decreased renal function (e.g., increased BUN, increased creatinine)	Hematuria
Digestive			Abdominal pain, nausea /

system			vomiting, gastritis, duodenal inflammation
Sensory organs	Headache, malaise		Dizziness, insomnia, dysgeusia, sensory hearing loss
Eye			Vitreous hemorrhage, conjunctivitis
Other	Shunt thrombosis / obstruction, increased LDH		Residual blood in dialysis circuit, musculoskeletal pain, shunt pain, fever, chest discomfort, edema, poor hemostasis, diabetic gangrene, hot flashes*

【Ver. 2.0⇒Ver. 2.1】

Created December 24, 2015

**Reason for change**	**Changes**	**Prior to change**	**After change**
To extend the registration period due to outstanding patients	0.1 Schema<br>0.5 Research period<br>11.2 Research period	Patient registration period: approval date–March 31, 2016	Patient registration period: approval date–September 30, 2016
Due to coordinator changes	7.2 Patient registration<br>23.3 BRIGHTEN Research Secretariat	BRIGHTEN Research Secretariat<br>Department of Nephrology and Endocrinology, University of Tokyo Graduate School of Medicine<br>Coordinator: Masaomi Nangaku, Takehiko Wada	BRIGHTEN Research Secretariat<br>Department of Nephrology and Endocrinology, University of Tokyo Graduate School of Medicine<br>Coordinator: Masaomi Nangaku, Hideki Kato

Due to coordinator changes	23.8 Study Group Advisors	Yoshiharu Tsubakihara Department of Comprehensive Kidney Disease Research, Osaka University Graduate School of Medicine	Yoshiharu Tsubakihara Graduate School of Health Care Sciences, Jikei Institute

【Ver. 2.1⇒Ver. 2.2】

Created February 7, 2018

<table>
<tr><th>Reason for change</th><th>Changes</th><th>Prior to change</th><th>After change</th></tr>
<tr><td>To add inspections necessary for achieving the objective of this research</td><td>List of inspection items</td><td><table><tr><th>Abbreviation</th><th>Inspection item</th></tr><tr><td>～</td><td>～</td></tr><tr><td>HbA1c</td><td>Hemoglobin A1c</td></tr><tr><td>LDH</td><td>Lactate dehydrogenase</td></tr><tr><td>MCV</td><td>Mean corpuscular volume</td></tr><tr><td>PCR</td><td>Protein creatinine ratio</td></tr><tr><td>～</td><td>～</td></tr></table></td><td><table><tr><th>Abbreviation</th><th>Inspection item</th></tr><tr><td>～</td><td>～</td></tr><tr><td>HbA1c</td><td>Hemoglobin A1c</td></tr><tr><td>LDH</td><td>Lactate dehydrogenase</td></tr><tr><td>MCP-1</td><td>Monocyte Chemotacic Protein-1</td></tr><tr><td>MCV</td><td>Mean corpuscular volume</td></tr><tr><td>PCR</td><td>Protein creatinine ratio</td></tr><tr><td>～</td><td>～</td></tr></table></td></tr>
<tr><td>For transfer of secretariat business</td><td>23.11 Implementation of business concluded in consigned research</td><td>BRIGHTEN MC&P Secretariat (inquiries regarding contracts, conference hosting)
MC&P Co., Ltd.</td><td>TRI BRIGHTEN Secretariat (inquiries regarding contracts, conference hosting)
Translational Research Informatics Center</td></tr>
</table>

<table>
<tr><td></td><td>contract with medical institutions</td><td></td><td></td></tr>
<tr><td>To add inspections necessary for achieving the objective of this research</td><td>0.8 Observation / inspection / reporting schedule

10.3 Observation / inspection / reporting schedule</td><td><table>
<tr><td>Time / Item</td><td>Registration</td><td>Prior to start of treatment</td><td colspan="11">Observation period</td><td>Simultaneous outcome</td><td>Discontinuation of participation / withdrawal of consent</td></tr>
<tr><td></td><td></td><td></td><td>Start of treatment</td><td>2, 4, 6, 8, 10 weeks</td><td>12 weeks</td><td>16 weeks</td><td>24 weeks</td><td>36 weeks</td><td>48 weeks</td><td>60 weeks</td><td>72 weeks</td><td>84 weeks</td><td>96 weeks</td><td></td><td></td></tr>
<tr><td>Blood test (central measurement): high-sensitivity CRP, folic acid, VB12, NT-proBNP, Fe, ferritin, TIBC</td><td></td><td></td><td>★</td><td></td><td>★</td><td></td><td></td><td></td><td></td><td></td><td></td><td></td><td>★</td><td></td><td></td></tr>
</table></td><td><table>
<tr><td>Time / Item</td><td>Registration</td><td>Prior to start of treatment</td><td colspan="11">Observation period</td><td>Simultaneous outcome</td><td>Discontinuation of participation / withdrawal of consent</td></tr>
<tr><td></td><td></td><td></td><td>Start of treatment</td><td>2, 4, 6, 8, 10 weeks</td><td>12 weeks</td><td>16 weeks</td><td>24 weeks</td><td>36 weeks</td><td>48 weeks</td><td>60 weeks</td><td>72 weeks</td><td>84 weeks</td><td>96 weeks</td><td></td><td></td></tr>
<tr><td>Blood test (central measurement): high-sensitivity CRP, folic acid, VB12, NT-proBNP, Fe, ferritin, TIBC, <u>MCP-1</u></td><td></td><td></td><td>★</td><td></td><td>★</td><td></td><td></td><td></td><td></td><td></td><td></td><td></td><td>★</td><td></td><td></td></tr>
</table></td></tr>
<tr><td>To add inspections necessary for achieving the objective of this research</td><td>10.1.3 Observation period (from start of treatment－96 weeks later)</td><td><table>
<tr><td rowspan="5">6</td><td rowspan="2">Blood test</td><td>Inspection date</td><td>yyyy/mm/dd</td></tr>
<tr><td>Inspection items</td><td>MCV, Hb (g/dL), serum Cr (mg/dL), eGFR (automatic calculation) (mL/min/1.73m^2), serum Alb (g/dL), serum iron (μg/dL), ferritin (ng/mL), TIBC or UIBC (μg/dL)</td></tr>
<tr><td>Blood test:</td><td colspan="2">(NGSP%) only diabetic patients measured at the start of treatment</td></tr>
<tr><td>HbA1c</td><td colspan="2">(inspections up to eight weeks prior to start of treatment allowed)</td></tr>
<tr><td>Blood test: central measurement (start, 12 weeks, 96 weeks)</td><td colspan="2">High sensitivity CRP, folic acid, Vitamin B12, NT-pro BNP, serum iron, ferritin, TIBC</td></tr>
</table></td><td><table>
<tr><td rowspan="5">6</td><td rowspan="2">Blood test</td><td>Inspection date</td><td>yyyy/mm/dd</td></tr>
<tr><td>Inspection items</td><td>MCV, Hb (g/dL), serum Cr (mg/dL), eGFR (automatic calculation) (mL/min/1.73m^2), serum Alb (g/dL), serum iron (μg/dL), ferritin (ng/mL), TIBC or UIBC (μg/dL)</td></tr>
<tr><td>Blood test:</td><td colspan="2">(NGSP%) only diabetic patients measured at the start of treatment</td></tr>
<tr><td>HbA1c</td><td colspan="2">(inspections up to eight weeks prior to start of treatment allowed)</td></tr>
<tr><td>Blood test: central measurement (start, 12 weeks, 96 weeks)</td><td colspan="2">High sensitivity CRP, folic acid, Vitamin B12, NT-pro BNP, serum iron, ferritin, TIBC, <u>MCP-1</u></td></tr>
</table></td></tr>
</table>

To add inspections necessary for achieving the objective of this research	10.2 Central measurement inspection	(omitted)<br>Samples collected by the central batch measurement inspection will be stored according to the SRL standard operating procedure after measurements are completed, after which they are to be stored for 10 years. Samples should be properly discarded after the end of the storage period. Additionally, if inspection items that are thought necessary to be added in order to achieve the objectives of this study following the start of this study, then the stored samples will be used for measurement after disclosing information by an appropriate method and after obtaining the approval of the principal investigator-affiliated facility, the Foundation for the Promotion of Advanced Medicine, and the ethical review committee of the research consignor. Stored samples and data will not be used for secondary purposes outside of the objectives of this study without permission from the subjects. The lead principal physician and study physician are to also obtain consent for sample storage and additional measurements with stored samples when consent is obtained from the patient for research participation.	(omitted)<br>Samples collected by the central batch measurement inspection will be stored according to the SRL standard operating procedure after measurements are completed, after which they are to be stored for 10 years. Samples should be properly discarded after the end of the storage period. Additionally, if there are inspection items that are thought necessary to be added in order to achieve the objectives of this study following the start of this study, then the stored samples will be used for measurement after disclosing information by an appropriate method and after obtaining the approval of the principal investigator-affiliated facility, the Foundation for the Promotion of Advanced Medicine, and the ethical review committee of the research consignor (*). Stored samples and data will not be used for secondary purposes outside of the objectives of this study without permission from the subjects. The lead principal physician and study physician are to also obtain consent for sample storage and additional measurements with stored samples when consent is obtained from the patient for research participation.<br><br>*Added February 2018<br>The steering committee decided to use stored samples (all subjects, planned for entire period) to measure MCP-1 at Kanazawa University.<br>SRL will dispense a part of the stored samples and transfer them

			to Kanazawa University according to the separately prepared operating procedure. SRL and Kanazawa University will create sample transfer records and appropriately store them. Kanazawa University will use the divided samples only for the measurement of MCP-1, and the remaining samples are immediately discarded after measurement. Information will be disclosed on the website of the Foundation for Advanced Medical Promotion and each implementing medical institution prior to implementation, and the information will not be used if the subject submits a refusal request for sample use.
Due to changes in affiliated institution names, transfers	23.4 Steering committee	Hideki Hirakata Division of Nephrology and Dialysis Center, Japanese Red Cross Fukuoka Hospital<br><br>Takashi Wada Department of Laboratory Medicine, Institute of Medical, Pharmaceutical and Health Sciences, Faculty of Medicine, Kanazawa University<br>Terumasa Hayashi Department of Kidney Disease and Hypertension, Osaka General Medical Center	Hideki Hirakata Fukuoka Renal Clinic<br>Takashi Wada Department of Nephrology and Laboratory Medicine, Institute of Medical, Pharmaceutical and Health Sciences, Faculty of Medicine, Kanazawa University<br>Terumasa Hayashi Department of Kidney Disease and Hypertension, Osaka General Medical Center
For exchange	23.5 Statistical analysis manager	Tatsuo Kagimura Translational Research Informatics Center	Kenichiro Tanabe Translational Research Informatics Center
To add inspection institutions	23.10 Blood test (central measurement) inspection institutions and sample collection / transportation	SRL Medisearch Inc.<br>President and CEO Hiroyuki Sekiguchi	① SRL Medisearch Inc.<br>President and CEO Hiroyuki Sekiguchi<br>Creation of materials needed for sample collection, collection of samples from medical institutions, storage of samples and

			distribution of samples to other central measurement institutions (dispensing, transfer), and inspection of the following items:<br>Inspection items: high sensitivity CRP, folic acid, Vitamin B12, NT-proBNP, serum iron, ferritin, TIBC<br><br>② Kanazawa University Hospital<br>Hospital Director Toshifumi Gabata<br>Implementation of inspections for the following items:<br>Inspection item: MCP-1

End
