# Supplementary material for: Hyporesponsiveness to erythropoiesis-stimulating agent in non-dialysis-dependent CKD patients: The BRIGHTEN study
Source: PLoS One. 2022 Nov 29;17(11):e0277921. doi: 10.1371/journal.pone.0277921 (PMC9707758; doi:10.1371/journal.pone.0277921)
Supplement: S2 File — (PDF) [file pone.0277921.s004.pdf]

oBservational clinical Research In chronic kidney disease patients with renal anemia: renal proGnosis in patients with Hyporesponsive anemia To Erythropoiesis stimulating agents, darbepoetiN alfa
BRIGHTEN

Research Protocol

Principal investigator : Department of Nephrology, Nagoya University Graduate School of Medicine Seiichi Matsuo
Co-principal investigator : Department of Nephrology and Endocrinology, University of Tokyo Graduate School of Medicine Masaomi Nangaku

Research support institution: Translational Research Informatics Center
Research consignor: Kyowa Hakko Kirin Co., Ltd.

May 2, 2014 Ver. 1.0 (approved by ethics review board of principal investigator facility)

All those involved in this research follow the spirit of the “World Medical Association Declaration of Helsinki” and “Ethical Guidelines for Clinical Research” (fully revised on July 31, 2008; Ministry of Health, Labor and Welfare).

Table of contents

0. Overview 5
1. Objective 9
2. Background and rationale 9
3. Drug information 11
4. Diagnostic criteria and disease stage / type classification 11
5. Eligibility criteria 13
6. Explanation and consent 13
7. Research participation procedure and patient registration 14
8. Treatment plan 16
9. Evaluation and reporting of adverse events 20
10. Observation / inspection / report items and schedule 24
11. Sample size and research period 32
12. Definition of endpoints 33
13. Statistical analysis 34
14. Input and submission of case report form 36
15. Research management 38
16. Various committees 38
17. Ethical items 38
18. Research costs and burdens 41
19. Amendment of Research Protocol 42
20. Research completion and early discontinuation 43
21. Record keeping 43
22. Publication of research and attribution of results 45
23. Research organization 46
24. References 48

List of abbreviations

Abbreviation	Non-abbreviated term
BFU-E	Burst-forming unit-erythroid
CFU-E	Colony-forming-unit-erythroid
CVD	Cardiovascular disease
CKD	Chronic kidney disease
KDIGO	Kidney disease: improving global outcomes
KDOQI	Kidney disease outcomes quality initiative
EPO	Erythropoietin（erythropoietin）
ERI	ESA response index
ESA	Erythropoiesis stimulating agent
HD	Hemodialysis

List of inspection items

Abbreviation	Inspection item
Alb	Albumin
Al-P	Alkaline phosphatase
ALT (GPT)	Alanine transaminase (glutamic pyruvate transaminase)
AST (GOT)	Aspartate amino transferase (glutamic oxaloacetic transaminase)
BNP	Brain natriuretic polypeptide
BUN	Blood urea nitrogen
Cr	Creatinine
CRP	C-Reactive protein
eGFR	Estimated glomerular filtration rate
Fe	Serum iron
γ-GTP	γ-glutamyl transpeptidase
Hb	Hemoglobin
HbA1c	Hemoglobin A1c
LDH	Lactate dehydrogenase
MCV	Mean corpuscular volume
PCR	Protein creatinine ratio
PTH	Parathyroid hormone
PWV	Pulse wave velocity
TIBC	Total iron binding capacity
TSAT	Transferrin saturation
UIBC	Unsaturated iron binding capacity

0. Overview

0.1 Schema

Subject patient selection
Chronic kidney disease patients who were diagnosed with renal anemia and are expected to be administered darbepoetin alfa

↓

Informed consent
This research will be sufficiently explained to patients deemed appropriate for this research, and written consent is obtained (see “6. Explanation and consent” for details).

↓

Eligibility confirmation
Patient eligibility will be determined based on “5. Eligibility criteria”.

↓

Registration
Sample size: 2,000<br>Registration period: approval date–March 31, 2016

↓

Observation period
Observation period: 96 weeks<br>Treatment: administer darbepoetin alfa according to package insert<br>Survey points: registration start of treatment–96 weeks and simultaneous outcome inspection<br>(implemented 96 weeks after the start of the last registered patient)

0.2 Objective

Investigate the actual conditions of patients with chronic kidney disease who were diagnosed with renal anemia and who received darbepoetin alfa (DA), and search for a new erythropoiesis stimulating agent (ESA) response index (ERI) relating to the expression of renal function deterioration and cardiovascular disease (CVD) events.

0.3 Eligibility criteria

Patients with chronic kidney disease and renal anemia who satisfy all the following selection criteria at the time of registration and for whom none of the exclusion criteria apply.

Selection criteria

1) Patients who are scheduled to start treatment with darbepoetin alfa within eight weeks after registration and who have not received ESA within 12 weeks prior to registration
2) Patients with an eGFR less than 60 mL/min/1.73 m^2 at the latest inspection within eight weeks prior to registration
3) Patients with an Hb concentration less than 11.0 g/dL at the latest inspection within eight weeks prior to registration
4) Patients aged over 20 years at the time of consent acquisition
5) Patients who have voluntarily provided written consent regarding participation in this research

Exclusion criteria

6) Patients who plan to initiate maintenance dialysis or have a kidney transplant within 24 weeks after registration
7) Patients with malignant tumors, blood disorders, or obvious hemorrhagic lesions
8) Patients with hypersensitivity to ESA components or ESA
9) Pregnant women, lactating women, patients who may be pregnant, or patients wishing to be pregnant during the research period
10) Patients participating in other clinical trials
11) Other patients who were deemed inappropriate for this research by the lead principal physician or study physician

0.4 Sample size

Sample size: 2,000

Predicted number of participating facilities: 200

0.5 Research period

Patient registration period: approval date–March 31, 2016

Observation period completion date: 96 weeks after the last registered patient (simultaneous outcome inspection)

0.6 Research design

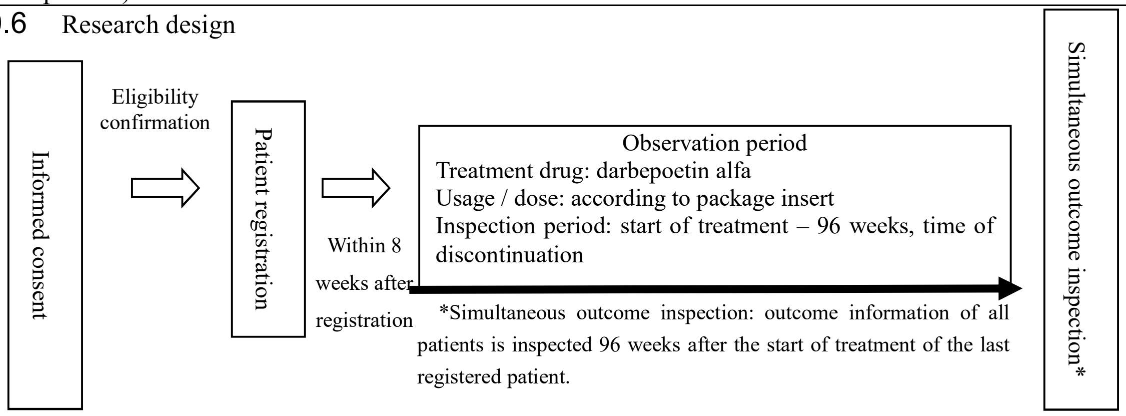

Research design: prospective observational trial   Implementing facilities: multi-center

0.7 Observation / inspection / reporting schedule

Observations, investigations, and inspections are conducted according to the following.

Item \ Time	Registration	Prior to start of treatment	Observation period: Start of treatment	Observation period: 2, 4, 6, 8, 10 weeks	Observation period: 12 weeks	Observation period: 16 weeks	Observation period: 24 weeks	Observation period: 36 weeks	Observation period: 48 weeks	Observation period: 60 weeks	Observation period: 72 weeks	Observation period: 84 weeks	Observation period: 96 weeks	Simultaneous outcome	Discontinuation of participation / withdrawal of consent
Eligibility determination	○														
Primary disease / complications / medical history		○													
PWV *1		○													
12-lead electrocardiogram / chest X-ray		●													
Height		○													
Weight			○	○	○	○	○	○	○	○	○	○	○		
Blood pressure			○		○		○		○		○		○		
Urinalysis: urine protein, urine Cr, PCR			○		○				○				○		
Blood test: HbA1c (only for diabetic patients) *2			○												
Blood test: Hb, MCV, Cr, eGFR, Alb, serum iron, ferritin, TIBC			○	○	○	○	○	○	○	○	○	○	○		
Blood test (central measurement): high-sensitivity CRP, folic acid, VB12, NT-proBNP, Fe, ferritin, TIBC			★		★								★		
Darbepoetin alfa administration status			→	→	→	→	→	→	→	→	→	→	→		

Iron formulation usage status			→	→	→	→	→	→	→	→	→	→	→		
Miscellaneous concomitant drug usage status			○	○	○	○	○	○	○	○	○	○	○		
Blood transfusion status			○	○	○	○	○	○	○	○	○	○	○		
Anemia influencing factor information			○	○	○	○	○	○	○	○	○	○	○		
Events / adverse events			→	→	→	→	→	→	→	→	→	→	→	→	→
Outcome information			→	→	→	→	→	→	→	→	→	→	→	→	→
Discontinuation / consent withdrawal															○
Inspection content			0	±1 week			±6 weeks								4 weeks
Case report form submission deadline	☆	☆			☆	☆	☆		☆		☆		☆	☆	
	After registration or within four weeks of end of final inspection of each case report form														

○ : Required

● : Implement before start of treatment if no inspection results within 12 weeks prior to registration. Do not record in case report form, and report when event occurs at the request of event evaluation committee.

◇ : Implement only for Hb, Cr, eGFR (when inspection is implemented within eight weeks prior to registration, its latest value is allowed).

★Central measurement: collect blood prior to darbepoetin alfa administration.

*1: Report latest inspection values from 12 weeks prior to registration to five weeks after registration.

*2: Report latest inspection values from eight weeks prior to treatment to start of treatment.

1. Objective

This purpose of this research is to investigate the actual conditions of patients with chronic kidney disease who were diagnosed with renal anemia and who received darbepoetin alfa (DA), to determine a new erythropoiesis-stimulating agent (ESA) response index (ERI) related to the poor prognosis of renal function and cardiovascular disease (CVD) events.

2. Background and rationale

Chronic kidney disease (CKD) is a very important disease worldwide. According to the Global Burden of Disease study in 2010, the annual age-adjusted mortality rate due to CKD in 1990 was 15.7 out of 100,000 people (27th leading cause of death), whereas that in 2010 was 16.3 out of 100,000 people (18th leading cause of death), and this rate is increasing annually[1]. Moreover, CKD is the 8th leading cause of death among Japanese people[2], and it is estimated that there were 13.3 million afflicted patients in 2006[3].

CKD is defined as a state in which renal damage (e.g., proteinuria) or decreased renal function with a glomerular filtration rate (GFR) of less than 60 mL/min/1.73 m^2 for over three months is present, and its severity is evaluated by the CGA classification of cause (Cause: C), renal function (GFR: G), and proteinuria (albuminuria: A). CKD treatment first involves lifestyle improvements (e.g., smoking cessation, salt reduction, and obesity improvements), after which antihypertensive drugs, such as angiotensin-converting enzyme (ACE) inhibitors and angiotensin receptor blockers, are used depending on the presence of diabetic complications and the extent of proteinuria[4]. CKD, which does not yet involve dialysis, is classified as pre-dialysis chronic kidney disease, and its treatment criteria differ from those involving hemodialysis (HD).

Erythropoiesis stimulating agents (ESAs), such as recombinant human erythropoietin (rHuEPO), are used for renal anemia treatment. ESA indications were also expanded to CKD patients in 1994, and various positive effects, such as quality of life (QoL) improvements have been reported, as well as dramatic reductions in blood transfusions, and a positive effect on vital prognosis after dialysis initiation[5].

The existence of “low ESA response” patient groups, where Hb concentrations do not increase much despite ESA administration, has become a problem in recent years. Iron deficiencies are thought to be the biggest cause of low ESA response, and other influential causes include bleeding, hematopoietic inhibition (e.g., infection, inflammation, folic acid/vitamin B12 deficiency), hematopoietic tumors, blood disorders, hypersplenism, appearance of anti-EPO antibodies, insufficient dialysis, and nutrient deficiency[5]. However, there is no clear definition for low ESA response in the current guidelines of renal anemia treatment in Japan and in Western countries. The definition of low ESA response in the “Guidelines for the treatment of renal anemia in patients with chronic kidney disease” included only the following: “In chronic kidney disease, a patient is considered to have a low response to rHuEPO therapy when rHuEPO is used subcutaneously once a week, with 6,000 units per session (6,000 units per week), and the target Hb concentration cannot be achieved (opinion). However, achieving the target Hb

concentration is difficult at the currently available doses of rHuEPO, so it is difficult to define a low response to rHuEPO therapy at the current stage"[5]. The current status is similar in international guidelines (KDIGO), which only state the following: "Cases where increases in Hb concentration from baseline values are not seen in the first month of ESA therapy regardless of the optimal dose in terms of body weight are classified as low ESA response (no grade)," and "Cases where doses are increased up to 50% of the necessary ESA administered dose during the period when Hb concentrations were stable two times are classified as low ESA response (no grade)"[6]. Under such circumstances, there is a need for the formulation of evidence-based indices of low ESA response and anemia treatment policies to treat CKD patients with renal anemia and low ESA response.

Large-scale clinical trials in the West on ESA treatment of CKD patients with renal anemia include the CHOIR trial[7], which used epoetin alfa and set deaths and cardiovascular disease (CVD) events at the treatment target Hb concentration from 2006 to 2009 as the outcome, and the TREAT trial[8], which used darbepoetin alfa. These trials initially reported that groups of CKD patients whose target Hb concentrations were set close to the reference values for healthy adults using ESA (> 13.0 g/dL) had a high CVD incidence rate, and that HB concentration normalization conversely increased the risk of CVD onset. However, subsequent sub-analysis showed that the prognosis of patients who achieved the target Hb concentration regardless of the magnitude of target Hb concentration settings was favorable[9]. Furthermore, the TREAT sub-analysis was conducted in 2010[10], which defined patients in the lower quartile of the Hb concentration change rate as the poor response group and all other patients as the favorable response group and conducted a comparative analysis of the two groups, and it showed that the poor ESA response group attained a lower average Hb concentration than the favorable response group, regardless of the higher ESA administered dose, and furthermore, that the poor ESA response group had a higher composite CVD endpoint occurrence rate and mortality rate than the favorable response group or placebo group. In other words, these results suggest that setting a high target Hb concentration itself is not the issue, but that the prognosis of patients with low ESA response is poor, or that the high ESA doses administered regardless of poor ESA response may have a negative impact. The above results indicate the importance of investigating not only the target Hb concentration but also the ESA response when conducting ESA treatment of CKD patients. However, whether background factors present in patients with a low ESA response increases the risk of CVD events or whether the administration of high concentrations of ESA is a problem is still debated, and further verification tests are needed. All large-scale clinical trial evidence on low ESA response and prognosis is based on Westerners, and there are no reports of large-scale evidence among Japanese people. It is assumed that the background factors and treatment conditions of CKD patients differ between the West and Japan[11]. Therefore, investigating the actual conditions of low ESA response among Japanese patients with CKD is important in investigating future treatment policies for patients with low ESA response.

Therefore, a multicenter prospective observational study of CKD patients who were not administered ESA and where darbepoetin alfa administration was planned was conducted with the research objective

of using darbepoetin alfa, which is an ESA that is widely used in renal anemia treatment among CKD patients with renal anemia, to collect a wide range of clinical information obtained from the initial stage of ESA treatment and investigate the actual conditions of patients with low response to darbepoetin alfa and factors associated with low response, as well as propose evaluation indices for low ESA response and cut-off values of related factors according to Japanese medical practice.

3. Drug information

A summary of the information on the drugs used in this research is described below. See the latest package insert, interview form, etc. for details.

darbepoetin alfa (generic name: darbepoetin alfa (genetic recombination)
Product name: NESP® Manufacturer: Kyowa Hakko Kirin Co., Ltd.)

1) Mechanism of action

Binds to erythropoietin receptors to promote the formation of colony-forming unit-erythroid (CFU-E) and burst-forming unit-erythroid (BFU-E)-derived colonies in a concentration-dependent manner in human bone marrow hematopoietic progenitor cells (*in vitro*).

2) Efficacy or effect

Renal anemia

3) Contraindications

Patients with hypersensitivity to components of this drug or erythropoietin-based drugs

4) Careful administration

(1) Patients with myocardial infarction, pulmonary infarction, cerebral infarction, or those with a history of such diseases and who are at risk of thromboembolism onset. It has been reported that blood viscosity increases with erythropoietin drugs and that there is a risk that thromboembolism may be exacerbated or induced, so sufficient observations should be made.
(2) Patients with hypertension (administration of this drug can cause hypertension and hypertensive encephalopathy could occur; therefore, sufficient observations should be made).
(3) Patients with a history of drug hypersensitivity.
(4) Patients with allergic predispositions.

4. Diagnostic criteria and disease stage / type classification

The diagnostic criteria and disease stage/type classification (definition) of the disease or condition specified in the eligibility criteria of this study are shown below.

Definition of chronic kidney disease[4]

Defined as (i), (ii), or both are sustained for over three months.

(i) Renal damage revealed by abnormal urine, diagnostic imaging, blood, and pathology. Proteinuria of over 0.15 g/gCr (albuminuria of over 30 mg/gCr) is particularly important

(ii) GFR ＜60 mL/min/1.73 m^2

Calculation equation of estimated GFR (eGFR)[4]

The estimated GFR (eGFR) is calculated using the following serum creatinine estimation equation (eGFRcreat):

Male eGFRcreat(mL/min/1.73 m^2) = 194 × $Cr^{-1.094}$ × $age^{-0.287}$

Female eGFRcreat(mL/min/1.73 m^2) = 194 × $Cr^{-1.094}$ × $age^{-0.287}$ × 0.739

Definition of renal anemia[5]

1) Renal anemia is defined as anemia caused by a decrease in EPO production ability in the kidney due to renal damage.

2) Renal anemia includes a shortened red blood cell lifespan, decreased EPO response of hematopoietic cells, malnutrition, and in-circuit residual blood in HD patients.

5. **Eligibility criteria**

Patients with chronic kidney disease and renal anemia who satisfy all the following selection criteria at the time of registration and for whom none of the exclusion criteria apply.

5.1 Selection criteria

1) Patients who are scheduled to start treatment with darbepoetin alfa within eight weeks after registration and who have not received ESA within 12 weeks prior to registration
2) Patients with an eGFR less than 60 mL/min/1.73 m^2 at the latest inspection within eight weeks prior to registration
3) Patients with an Hb concentration less than 11.0 g/dL at the latest inspection within eight weeks prior to registration
4) Patients aged over 20 years at the time of consent acquisition
5) Patients who voluntarily provide written consent regarding participation in this research

5.2 Exclusion criteria

1) Patients who plan to initiate maintenance dialysis or have a kidney transplant within 24 weeks after registration
2) Patients with malignant tumors, blood disorders, or obvious hemorrhagic lesions
3) Patients with hypersensitivity to ESA components or to ESA
4) Pregnant women, lactating women, patients who may be pregnant, or patients wishing to become pregnant during the research period
5) Patients participating in other clinical trials
6) Other patients deemed inappropriate for this research by the lead principal physician or study physician

6. **Explanation and consent**

The lead principal physician or study physician will sufficiently explain the research content to patients thought to be appropriate as subjects of this research based on explanations/consent documents set at each facility prior to participating in this research. Research collaborators can provide supplementary explanations for this research at this time. After giving patients sufficient time to consider whether to participate in the study, the lead principal physician or study physician will obtain the patient's voluntary consent to participate in the research in writing.

The lead principal physician or study physician who provided the explanation as well as the patient will write their name and provide their seal on the consent document or sign it, and each individual will write the date. Research collaborators who provide supplementary explanations will do the same.

If the individual cannot sign due to difficulties in writing, verbal consent will be obtained by the individual, and their representative will provide written consent. At that time, the date on which the

representative provided written consent, relationship with the patient, reason for consent by the representative (e.g., difficulties in writing), and date on which the patient provided verbal consent will be recorded on the consent document.

The lead principal physician or study physician will provide a copy of the consent form for the patient's records as well as an explanatory document to the patient. The lead principal physician or study physician will also store the consent documents for research in the medical institution's records along with the treatment records.

When there are issues relating to the decision to continue participation in this research, the lead principal physician or study physician will revise the explanatory document, provide explanations to patients participating in the research using the revised explanatory document, and obtain voluntary written consent from the individual regarding the continuation of participation in this research.

If possible, a withdrawal letter is created when a patient participating in the research submits a withdrawal of consent. The patient will write their name and provide their seal or sign as well as write the date of consent withdrawal, and the lead principal physician or study physician will write their name and provide their seal or sign the confirmation date. The lead principal physician or study physician will provide a copy of the signed and sealed consent withdrawal form to the patient, and the original consent withdrawal form will be stored at the applicable medical institution.

7. Research participation procedure and patient registration

7.1 Research participation procedure

A central registration system at the TRI data center in the Translational Research Informatics Center (henceforth, "data center") will be used to register for participation in this research. Facility registration and participating physician registration will be conducted using the following procedure.

7.1.1 Facility registration

1) The lead principal physician will send a copy of the affiliated facility ethics review board (Note) approval notification form to the BRIGHTEN TRI secretariat in the Translational Research Informatics Center by FAX.
Note: Another committee is acceptable if they are a committee that would examine the scientific validity, ethical validity, etc. of this research.

2) The BRIGHTEN TRI secretariat will submit the information of the facilities judged to be able to participate based on the obtained materials to the data center.

3) The data center will conduct facility registration and send a facility registration completion notification form to the lead principal physician.

4) The lead principal physician will confirm the eClinical Base website URL and initial password* for user account authentication described in the facility registration completion notification form.
*Initial password for user account authentication: temporary password used when setting the user

ID and password.

7.1.2 User registration

1) The lead principal physician or study physician will access the eClinical Base website URL recorded on the facility registration completion notification form, enter the necessary information on the website, and apply it to the user.

2) After applying for the user, the lead principal physician or study physician will print out the self-signed confirmation form, sign it, and send this to the data center by FAX.

3) The data center will e-mail a "temporary user account URL" for setting the user ID and password to the lead principal physician or study physician who applied for the user.

4) The lead principal physician or study physician will set a user ID and password online.

User registration is conducted using the same procedure under the supervision of the lead principal physician when the research collaborators input case report form data.

7.2 Patient registration

A central registration system at the data center is used for patient registration. Patient registration is conducted using the following procedure:

1) The lead principal physician or study physician will conduct the necessary inspections for determining the eligibility of patients whose participation in this research is deemed possible.

2) The lead principal physician or study physician will access the eClinical Base website URL and input patient information on the website. The patient identification code used at this time will not include individual information at each facility, and a unique and arbitrary code is used.

3) Observations according to protocol will be implemented when deemed eligible according to eligibility criteria.

4) An "anonymization number comparison table," etc. will be created at each facility to ensure that there is no misidentification of patients, and the lead principal physician of each facility or a representative will appropriately store and manage the anonymization number comparison table.

5) The lead principal physician or study physician will input case report form data on the website after patient registration. However, research collaborators can input this under the supervision of a lead principal physician.

6) The input and input deadline of the case report form will follow the "Input and submission of case report form." Questions regarding the input method should be directed to the data center.

8. Treatment plan

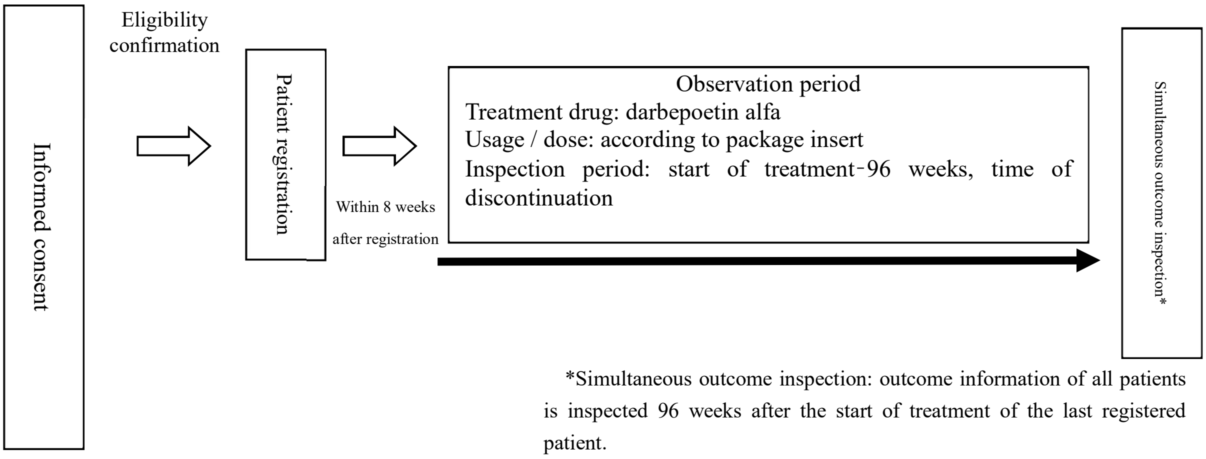

*Simultaneous outcome inspection: outcome information of all patients is inspected 96 weeks after the start of treatment of the last registered patient.

8.1 Recommended renal anemia treatment

This research is an observational study, so the following treatment methods are recommended.

Among CKD patients diagnosed with renal anemia, those for whom ESA administration was deemed necessary will receive the expected DA according to the package insert and observed for 96 weeks from the start of treatment (see Section 8.1.1 for target values of renal anemia treatment).

The participants in this study will include patients who did not receive ESA within 12 weeks prior to registration, and inspections in this research will be implemented following consent acquisition. DA administration will begin within eight weeks after registration.

The final administration of DA during the observation period will be performed immediately before 96 weeks after the start of treatment, but treatment will be continued as necessary after the completion of the observation period.

8.1.1 Target values of renal anemia treatment

Refer to the latest information, such as the guidelines of academic societies, etc., for the target Hb concentration in ESA therapy.

8.1.2 Recommended darbepoetin alfa administration method

The following administration method is recommended for darbepoetin alfa according to the guidelines and latest package inserts.

Table Target Hb concentrations of each guideline and the Hb concentration at the start of treatment

Guideline	Target Hb concentration	Hb concentration at the start of treatment
2008 Japanese Society for	Over 11 g/dL. Consider dose reduction / suspension if over 13 g/dL*1.	Less than 11 g/dL

Dialysis Therapy		
2012 CKD clinical guideline	10-12 g/dL. Take measures not to exceed 12 g/dL. Do not exceed 13 g/dL.	Less than 10 g/dL
2012 KDIGO guideline	10-11.5 g/dL*2. Do not exceed 13 g/dL.	Less than 10 g/dL*3

* 1 However, consider dose reduction/suspension for patients already with or who have complications of serious CVD or those with medical needs when the Hb concentration exceeds 12 g/dL.

* 2 Quality of life (QoL) may improve in certain patient groups even when the Hb concentration exceeds 11.5 g/dL and makes the risk acceptable, so it is thought that treatment according to each individual patient is needed.

* 3 QoL may improve in certain patient groups at even higher Hb concentrations, and ESA therapy may start at a stage of >10 g/dL, so treatment according to each individual patient is valid.

(Reference: "Evidence-based CKD practice guideline 2013")

(refer to the package insert, 2008 edition of the "Guidelines for the treatment of renal anemia in patients with chronic kidney disease" by the Japanese Society for Dialysis Therapy)

The package insert (excerpt) is shown below.

【Dosage and administration】

- Initial dose

  Adult: A usual adult dose of 30 μg of darbepoetin alfa (genetic recombination) per session is administered subcutaneously or intravenously once every two weeks.

- Maintenance dose

  Adult: When improved effects are seen for anemia, a usual adult dose of 30–120 μg of darbepoetin alfa (genetic recombination) per session is administered subcutaneously or intravenously once every two weeks. When anemia improvement is maintained with an administration once every two weeks, then the starting dose is twice the dose at that time; dosing frequency is changed to once every four weeks, and 60–180 μg per session can be administered subcutaneously or intravenously once every four weeks.

  The dose may be adjusted according to the extent of anemic symptoms, age, etc., but the maximum administered dose is set at 180 μg per session.

【Dose adjustment】

The dose should be adjusted with reference to Table 8.2-1 in cases where dose adjustments are necessary, such as when appropriate increases in the Hb concentration or hematocrit value are not observed in the initial administration period, or when the Hb concentration or hematocrit

value deviated from the target range for two consecutive weeks during the maintenance administration period. When increasing doses, do so one step at a time as a general rule.

The table below shows the dose adjustment table for subcutaneous administration. The latest package insert for intravenous injections was followed.

Table 8.2-1 Dose adjustment table for adults (during subcutaneous administration)

Step	Administered dose of this drug
1	15 μg
2	30 μg
3	60 μg
4	90 μg
5	120 μg
6	180 μg

【When changing administration level】

1) When changing the administration interval of this drug, shifts in the Hb concentration or hematocrit value will be sufficiently observed prior to extending the administration interval to confirm that stable shifts in the Hb concentration or hematocrit value are shown at the same administered dose. After doing so, change from once a week to once every two weeks, or once every two weeks to once every four weeks. Confirm shifts in the Hb concentration or hematocrit value after the change and adjust as appropriate.

2) Patients in whom the Hb concentration or hematocrit value did not reach the target range even after administering 180 μg per session should change the administered dose to 1/2 and change the administration frequency from once every two weeks to once every week, or once every four weeks to once every two weeks.

8.2 Discontinuation criteria for each patient and its handling

The necessary items are recorded up to the traceable time point in the case report form and the “discontinuation report form” in the following patients.

1) When a patient has died.

2) When a patient cannot be contacted and becomes untraceable.

However, if the transfer destination is known, the patient will be tracked by obtaining data from the transfer destination facility or by calling, and tracing will be continued.

3) When a patient requests discontinuation from research participation (patient information prior to discontinuation can be used).

4) When a patient has requested consent withdrawal (patient information after registration cannot be used).

5) When the lead principal physician or study physician deemed that research participation should be discontinued.

Patients in whom any of the following are applicable are not considered discontinued, with normal observation period inspections continued to the extent possible, and information up to the time point of 96 weeks collected.

1) Patients who transitioned to renal replacement therapy (dialysis, kidney transplantation).
2) When a patient has requested discontinuation of darbepoetin alfa use.
3) When the lead principal physician or study physician deemed that darbepoetin alfa use should be discontinued due to adverse events, etc.

The lead principal physician or study physician will make efforts to provide the best possible treatment to the patient even after discontinuation of research participation.

If it was determined after registration that a patient did not meet the eligibility criteria at the time of registration, then this will be considered “ineligible after registration,” and research participation will be concluded for the patient. Adverse event information up to 30 days after the decision to complete participation will be reported for patients in whom darbepoetin alfa was administered even once.

8.3 Recommended concomitant treatment

8.3.1 Iron replenishment therapy

The presence of iron is important for the development of the improvement effects of darbepoetin alfa on renal anemia, and iron formulations should be administered with reference to the latest information, such as the guidelines of academic societies when iron efficiency occurs.

Table Iron administration criteria of each guideline

Guideline	Iron formulation administration criteria
2006 KDOQI guideline	Replenish so as to maintain ferritin value ＞100 ng／mL and TSAT ＞20％. However, there is little evidence for administering iron formulations at ferritin value ＞500 ng/mL.
2008 Japanese Society for Dialysis Therapy	Replenish iron during ESA therapy when TSAT is less than 20% and ferritin value is less than 100 ng/mL. *1
2012 CKD clinical guideline	Replenish iron during ESA therapy when TSAT is less than 20% and ferritin value is less than 100 ng/mL. Do not intentionally increase when ferritin value is above 250 ng/mL.
2012 KDIGO guideline	Hb increases or ESA decreases are desirable in renal anemia patients who are not using iron formulations, and iron formulations should be administered if TSAT ≦30％ and ferritin value ≦500 ng/mL.

*1 The upper limits of TSAT and ferritin values are not specified.

(reference: “evidence-based CKD clinical guideline 2013”).

8.3.2 Treatment where concomitant use is prohibited

The administration of drugs of the same type or with the same efficacy as darbepoetin alfa (e.g., epoetin beta pegol, epoetin alfa, epoetin beta) is prohibited during the observation period.

If prohibited concomitant use is applied during the observation period, the reason is recorded in the case report form, and research participation is continued.

8.4 Post-treatment

No particular provisions are made regarding treatment after the discontinuation/completion of this research.

The lead principal physician or study physician will make efforts to provide appropriate and optimal treatments according to guidelines even after the discontinuation/completion of the research or its early discontinuation (see “20. Research completion and early discontinuation”).

9. **Evaluation and reporting of adverse events**

9.1 Definition

9.1.1 Definition of adverse event

An adverse event refers to any unfavorable medical event (e.g., subjective and objective symptoms, abnormal laboratory test values) observed during or within 30 days after the discontinuation or termination of administration of darbepoetin alfa, regardless of a causal relationship with darbepoetin alfa.

9.1.2 Definition of serious adverse events

The following are referred to as serious adverse events.

(1) Death

(2) Injury

(3) Risk of death

(4) Risk of injury

(5) Hospitalization or its extension due to treatment

(6) Serious events relating to above-mentioned (1)–(5) (other medically important conditions)

(7) Congenital disorders or abnormalities in later generations

9.1.3 Definition of outcome

The outcomes of adverse events in this study were determined based on the following definitions:

Recovery	When the adverse event has disappeared, and the patient has recovered to their state prior to the occurrence of the adverse event
Remission	When the adverse event has almost disappeared, and the patient has returned to a state similar to that prior to the occurrence of the adverse event
Non-recovery	When the adverse event has not disappeared, and the patient is in a state similar to that during the occurrence of the adverse event
Recovery but with	When the adverse event has disappeared, but the patient has sequelae

sequelae	
Death	When the lead principal physician or study physician determine that there is an association between the adverse event and death, or when its possibility cannot be ruled out
Unknown	When there is no information, and it is unknown

9.2 Evaluation and reporting of adverse events in this study

All adverse events that occurred during or within 30 days after discontinuation or termination of administration of darbepoetin alfa, all adverse events that occurred by the end of the simultaneous outcome survey are subject to evaluation in this study.

Serious adverse events, as specified in “9.1.2 Definition of serious adverse events” are reported according to “9.4 Emergency reporting of adverse events and subsequent responses” and “Appendix 2 Reporting and response manual when serious adverse event occurs.”

9.3 Expected adverse events

The side effects of the drugs studied in this research are as follows. Refer to the package insert for the latest information.

Darbepoetin alfa

<Adults>

In Japanese clinical trials, side effects (including abnormal laboratory test values) were observed in 471 of 1462 patients (32.2%). The main side effects were hypertension in 248 patients (17.0%), shunt thrombosis/obstruction in 44 patients (3.0%), headache in 28 patients (1.9%), and malaise in 20 patients (1.4%) (at the time of approval of NESP ® injection).

(1) Serious side effects

Cerebral infarction (0.9%): Cerebral infarction could occur, so sufficient observations should be made, and appropriate measures, such as discontinuing administration, should be implemented when abnormalities are observed.

Cerebral hemorrhage (0.1%): Cerebral hemorrhage could occur, so sufficient observations should be made, and appropriate measures, such as discontinuing administration, should be implemented when abnormalities are observed.

Hepatic dysfunction, jaundice (0.1%): Hepatic dysfunction or jaundice associated with increases in ALT (GPT) or γ-GTP could occur, so sufficient observations should be made, and appropriate measures, such as discontinuing administration, should be implemented when abnormalities are observed.

Hypertensive encephalopathy (incidence unknown): Hypertensive encephalopathy could occur; therefore, the drug should be administered while carefully monitoring shifts in blood pressure.

Shock and anaphylaxis (incidence unknown): Shock and anaphylaxis (e.g., urticaria, dyspnea, lip edema, pharyngeal edema) could occur, so sufficient observations should be made, and appropriate measures, such as discontinuing administration, should be implemented when abnormalities are observed.

Pure red cell aplasia (incidence unknown): Pure red cell aplasia accompanying anti-erythropoietin antibody production could occur; therefore, in this case, administration should be discontinued, and appropriate treatment should be conducted.

Myocardial infarction and pulmonary infarction (incidence unknown): Myocardial infarction and pulmonary infarction could occur, so sufficient observations should be made, and appropriate measures, such as discontinuing administration, should be implemented when abnormalities are observed.

(2) Other side effects

The following side effects could occur, so sufficient observations should be made, and appropriate measures, such as dose reduction/drug discontinuation, should be implemented when abnormalities are observed.

*: Frequency unknown

	Frequency of side effect (%)		
	Over 1%	0.5%–1%	Less than 0.5% or unknown frequency
Cardiovascular system	Hypertension	Arrhythmia	Hypotension during dialysis, palpitations, angina pectoris / myocardial ischemia, arteriosclerosis obliterans
Skin		Pruritus, rash	
Liver		Liver dysfunction, increased Al-P	Increased γ-GTP, increased AST (GOT), increased ALT (GPT), gallbladder polyps
Metabolism			Decreased stored iron, increased serum potassium, increased blood phosphorus, increased uric acid, decreased appetite, secondary hyperparathyroidism
Blood	Eosinophilia		Thrombocytopenia, leukocytosis, lymphopenia, leukopenia
Kidney / urinary system		Decreased renal function (e.g., increased BUN, increased creatinine)	Hematuria
Digestive system			Nausea / vomiting, abdominal pain, gastritis, duodenal inflammation
Sensory organs	Headache, malaise		Dizziness, sensory hearing loss

Eye			Vitreous hemorrhage, conjunctivitis
Other	Shunt thrombosis / obstruction, increased LDH		Residual blood in dialysis circuit, chest discomfort, shunt pain, poor hemostasis, diabetic gangrene, fever, musculoskeletal pain, hot flashes*

9.4 Emergency reporting of serious adverse events and subsequent responses

9.4.1 Adverse events which must be reported

Adverse events that must be reported include all serious adverse events specified in "9.1.2 Definition of serious adverse events," that occur from the start of darbepoetin alfa administration to within 30 days after its completion or discontinuation.

9.4.2 Reporting procedure

After confirming the occurrence of serious adverse events, the lead principal physician or study physician of each implementing medical institution will promptly list them in the "adverse events" section of the case report form of the online patient registration/report system. Serious adverse events input in the online report form system are reported to the principal investigator, etc., through the online system. Refer to "Appendix 2 Reporting and response manual when serious adverse events occur" for details of the procedure.

9.4.3 Correspondence procedure

The principal investigator and the BRIGHTEN Research Secretariat determine evaluations, recommendations, and countermeasures by the independent data monitoring committee and report to participating facilities as needed. Refer to "Appendix 2 Reporting and response manual when serious adverse events occur" for details of the procedure.

10. Observation / inspection / report items and schedule

10.1 Observation / inspection items

The items to be investigated in this research are shown below.

Observations and inspections of the following items will be conducted from the start of this research to the completion or discontinuation of the research, and the results will be recorded online in the case report form. The lead principal physician or study physician conducts the patient registration. After registration, research collaborators are allowed to record these under the supervision of the lead principal physician or study physician.

10.1.1 At the time of registration

The following items are investigated at the time of eligibility confirmation and recorded in the case report form.

<table>
<tr><td rowspan="4">1</td><td rowspan="4">Patient information</td><td>Date of birth</td><td>yyyy/mm/dd</td></tr>
<tr><td>Gender</td><td>Male, female</td></tr>
<tr><td>Date of consent acquisition</td><td>yyyy/mm/dd</td></tr>
<tr><td>Patient identification code</td><td>Record any code that does not include individual information
(refer to Appendix 1 How to use anonymization number comparison table)</td></tr>
<tr><td>2</td><td>Eligibility determination</td><td colspan="2">Selection criteria (5.1) / exclusion criteria (5.2) determination result (yes/no)</td></tr>
<tr><td rowspan="2">3</td><td rowspan="2">Blood test*</td><td>Inspection date</td><td>yyyy/mm/dd</td></tr>
<tr><td>Inspection items</td><td>Hb (g/dL), serum Cr (mg/dL), eGFR (automatically calculated) (mL/min/1.73 m^2)
eGFR is calculated using the following estimation equation.
Male: $194 \times Cr^{-1.094} \times age^{-0.287}$
Female: $194 \times Cr^{-1.094} \times age^{-0.287} \times 0.739$</td></tr>
</table>

*Provide the latest inspection values within eight weeks prior to registration.

10.1.2 Prior to start of treatment

The latest information up to the start date of treatment is provided as baseline data.

<table>
<tr><td rowspan="2">1</td><td rowspan="2">Primary disease</td><td>Disease name</td><td>Chronic glomerulonephritis, nephrosclerosis, multiple cystic kidneys, chronic nephritis, diabetic nephropathy, lupus nephritis, other (details)</td></tr>
<tr><td>Disease duration</td><td>X months, unknown</td></tr>
<tr><td>2</td><td>History / complications</td><td>Disease name (multiple choices allowed)</td><td>Stroke (including ischemic, hemorrhagic), ischemic heart disease, heart failure requiring hospitalization, other CVD, peripheral vascular disease, hypertension, diabetes, dyslipidemia, collagen disease and rheumatic disease, existing malignant tumor (including blood diseases), other diseases of note (details)</td></tr>
<tr><td>3</td><td>Smoking habits</td><td colspan="2">Current (currently smoking), former (smoked in the past but no longer smoking), never (never smoked)</td></tr>
<tr><td>4</td><td>Height</td><td colspan="2">XX.X cm</td></tr>
</table>

5	PWV	Right side, left side, average (cm/sec)<br>(provide latest information with data from 12 weeks prior to registration to five weeks after registration)
6	12-lead ECG	Implement before start of treatment if there are no inspection results within 12 weeks prior to registration. Do not record in case report form, and report at the request of the event evaluation committee at the time of event occurrence.
7	Chest X-ray	

10.1.3 Observation period (from start of treatment-96 weeks later)

Report the information closest to each survey point among the inspection results included in the permissible range.

Start of treatment: day of blood tests; urine tests can be performed up to -4 weeks, blood test HbA1c up to -8 weeks)

Weeks 2, 4, 6, 8, 10, 12, and 16 after the start of treatment: ± 1 week

Weeks 24, 36, 48, 60, 72, 84, 96 after the start of treatment: ± 6 weeks

1 *	Darbepoetin alfa administration status	Administration date	yyyy/mm/dd	
		Administration route	Intravenous administration, subcutaneous administration, other (details)	
		Administered dose	(μg)	
		Administered dose restriction	Presence	Present, absent
			Reason	Medical reason, financial reason, other
		Target Hb concentration (lower limit value)	(g/dL)	
2 *	Iron administration status	Administration presence	Present, absent	
		Oral medicine	Drug name	Ferrum, Incremin, Fero-Gradumet, Slow-Fe, Tetucur, Ferromia, Fenilene, Fenelmin, Feredaim, Ferrostec, Ferotym, Ferrofiel, Foliromin
			Prescribed date	yyyy/mm/dd
			Number of prescribed days	XX (days)
			Daily dose	mg
		Intravenous injection	Drug name	Fesin, Ferricon, other (drug name)
			Administration date	yyyy/mm/dd
			Administered dose	Daily dose as iron (mg)
3	Miscellaneous concomitant drug usage status	Immunosuppressants	Administration present, absent, unknown	
		Steroids	Administration present, absent, unknown	
		Antihypertensive drugs	Diuretics, calcium channel blockers, ACE inhibitors, angiotensin II receptor blockers, direct renin inhibitors, sympathetic blockers, α 2 receptor stimulants, other	
		Hypoglycemic drugs	Biguanide agents, thiazolidine agents, DPP-4 inhibitors, sulfonylurea agents, fast-acting insulin secretagogues, α -glucosidase inhibitors, insulin, SGLT2 inhibitors, other	
		Drugs where	Drug	Epoetin beta pegol, epoetin alfa, epoetin

<table>
<tr><td rowspan="2"></td><td rowspan="2"></td><td colspan="2" rowspan="2">concomitant use is prohibited</td><td>name</td><td>beta</td></tr>
<tr><td>Reason for use</td><td>[Written accordingly]</td></tr>
<tr><td>4</td><td>Weight</td><td colspan="4">XX.X (kg)</td></tr>
<tr><td>5</td><td>Blood pressure (start, 12, 24, 48, 72, 96 weeks)</td><td colspan="4">Diastolic blood pressure, systolic blood pressure (mmHg)</td></tr>
<tr><td rowspan="4">6</td><td rowspan="2">Blood test</td><td>Inspection date</td><td colspan="3">yyyy/mm/dd</td></tr>
<tr><td>Inspection items</td><td colspan="3">MCV, Hb (g/dL), serum Cr (mg/dL), eGFR (automatic calculation) (mL/min/1.73m^2), serum Alb (g/dL), serum iron (μg/dL), ferritin (ng/mL), TIBC or UIBC (μg/dL)</td></tr>
<tr><td>Blood test: HbA1c</td><td colspan="4">(NGSP%) only diabetic patients measured at the start of treatment (inspections up to eight weeks prior to start of treatment allowed)</td></tr>
<tr><td>Blood test: central measurement (start, 12 weeks, 96 weeks)</td><td colspan="4">High sensitivity CRP, folic acid, Vitamin B12, NT-pro BNP, serum iron, ferritin, TIBC</td></tr>
<tr><td>7</td><td>Urinalysis (start, 12, 48, 96 weeks)</td><td colspan="4">Urine protein (mg/dL), urinary Cr (mg/dL), PCR (protein creatinine ratio (g/Cr): automatically calculated)</td></tr>
<tr><td rowspan="2">8</td><td rowspan="2">Blood transfusion</td><td>Blood transfusion date</td><td colspan="3">yyyy/mm/dd</td></tr>
<tr><td>Blood transfusion amount</td><td colspan="3"></td></tr>
<tr><td>9</td><td>Development of factors that affect anemia</td><td colspan="4">Malignant tumors, hemorrhagic lesions, collagen disease and rheumatic disease, immunosuppressant or steroid use (apply “2. Concomitant drug usage status” data correspondingly), other myelosuppressive factors (details)
Note) refer to attachment for definition</td></tr>
<tr><td rowspan="4">10*</td><td rowspan="4">Event
Note) refer to attachment for definition</td><td rowspan="2">Event name
CVD event</td><td>Fatal</td><td colspan="2">Death due to myocardial infarction, sudden death, congestive heart failure, arrhythmia, cerebrovascular death, aortic dissection, ischemia of major organs other than the heart / brain, or other types of cardiovascular death</td></tr>
<tr><td>Non-fatal</td><td colspan="2">Myocardial infarction*1, hospitalization due to angina pectoris, ischemic heart disease requiring invasive intervention treatment, congestive heart failure with chronic organic heart disease*2 (excluding arrhythmia), severe arrhythmia and atrial fibrillation / atrial flutter, cerebrovascular disease, chronic arteriosclerosis obliterans, aortic dissection, or ischemia of major organs other than the heart / brain</td></tr>
<tr><td>Event name
Renal function deterioration</td><td colspan="3">Initiation of maintenance dialysis, kidney transplantation, 50% decrease in eGFR, eGFR less than or equal to 6.0 mL/min/1.73m^2</td></tr>
<tr><td>Onset date</td><td colspan="3">yyyy/mm/dd (date of initial diagnosis when unknown)</td></tr>
<tr><td rowspan="3">11*</td><td rowspan="3">Adverse event</td><td>Event name</td><td colspan="3">[Written accordingly]</td></tr>
<tr><td>Occurrence date</td><td colspan="3">yyyy/mm/dd</td></tr>
<tr><td>Severity</td><td colspan="3">Severe, not severe (refer to 9.1.2 Definition of serious adverse</td></tr>
</table>

			events)
		Causal relationship with darbepoetin alfa	Present, absent
		Outcome	Recovery, remission, non-recovery, recovery but with sequelae, death, unknown
		Outcome confirmation date	yyyy/mm/dd
12	Outcome	Outcome status	Survival, death (cause of death: fatal CVD event, other (details)), untraceable (reason)
		Confirmation date	Final confirmed survival date, death date

* Provide status from the previous survey point to the current survey point. Provide information two weeks prior at the start of treatment.

*1: New-onset (asymptomatic) myocardial infarction is treated as an event, even without hospitalization.

*2: Includes old myocardial infarction, valvular disease, epicarditis, etc.

10.1.4 Simultaneous outcome survey (96 weeks from the start of the final registered patient)

A simultaneous outcome survey will be conducted for all patients 96 weeks after the start of the final registered patient.

1	Outcome	Outcome status	Survival<br>Death (cause of death: fatal CVD event, other (details))<br>Unknown (reason)	
		Confirmation date	Final confirmed survival date, death date	
2*	Event Note) refer to attachment for definition	Event name CVD event	Fatal	Death due to myocardial infarction, sudden death, congestive heart failure, arrhythmia, cerebrovascular death, aortic dissection, ischemia of major organs other than the heart / brain, or other types of cardiovascular death
			Non-fatal	Myocardial infarction*1, hospitalization due to angina pectoris, ischemic heart disease requiring invasive intervention treatment, congestive heart failure with chronic organic heart disease*2 (excluding arrhythmia), severe arrhythmia and atrial fibrillation / atrial flutter, cerebrovascular disease, chronic arteriosclerosis obliterans, aortic dissection, or ischemia of major organs other than the heart / brain
		Event name Renal function deterioration	Initiation of maintenance dialysis, kidney transplantation, 50% decrease in eGFR, eGFR less than or equal to 6.0 mL/min/1.73m^2	
		Onset date	yyyy/mm/ dd (date of initial diagnosis when unknown)	
3*	Adverse event	Event name	[Written accordingly]	
		Occurrence date	yyyy/mm/dd	
		Severity	Severe, not severe (refer to 9.1.2 Definition of serious adverse events)	
		Causal	Present, absent	

		relationship with darbepoetin alfa	
		Outcome	Recovery, remission, non-recovery, recovery but with sequelae, death, unknown
		Outcome confirmation date	yyyy/mm/dd

* Provide the status from the previous survey point to the simultaneous outcome survey time point.

*1: New-onset (asymptomatic) myocardial infarction is treated as an event, even without hospitalization.

*2: Includes old myocardial infarction, valvular disease, epicarditis, etc.

10.1.5 When discontinuing / withdrawing consent for participation in research

Provide answers within four weeks of discontinuation or consent withdrawal from research participation. Items from two onwards are not needed for patients who withdraw consent. Investigations during the normal observation period are continued if the patient can be traced even after the discontinuation of darbepoetin alfa administration or after event occurrence.

1	Research participation discontinuation / consent withdrawal	Discontinuation / consent withdrawal date	yyyy/mm/dd	
		Reason for discontinuation	Death, untraceable, patient request, consent withdrawal, physician decision, other (reason)	
2	Outcome	Outcome status	Survival, death (cause of death: fatal CVD event, other (details)), unknown (reason)	
		Confirmation date	Final confirmed survival date, death date	
4*	Event Note) refer to attachment for definition	Event name CVD event	Fatal	Death due to myocardial infarction, sudden death, congestive heart failure, arrhythmia, cerebrovascular death, aortic dissection, ischemia of major organs other than the heart / brain, or other types of cardiovascular death
			Non-fatal	Myocardial infarction*1, hospitalization due to angina pectoris, ischemic heart disease requiring invasive intervention treatment, congestive heart failure with chronic organic heart disease*2 (excluding arrhythmia), severe arrhythmia and atrial fibrillation / atrial flutter, cerebrovascular disease, chronic arteriosclerosis obliterans, aortic dissection, or ischemia of major organs other than the heart / brain
		Event name Renal function deterioration	Initiation of maintenance dialysis, kidney transplantation, 50% decrease in eGFR, eGFR less than or equal to 6.0 mL/min/1.73m^2	
		Onset date	yyyy/mm/dd (date of initial diagnosis when unknown)	

5*	Adverse event	Event name	[Written accordingly]
		Occurrence date	yyyy/mm/dd
		Severity	Severe, not severe (refer to 9.1.2 Definition of serious adverse events)
		Causal relationship with darbepoetin alfa	Present, absent
		Outcome	Recovery, remission, non-recovery, recovery but with sequelae, death, unknown
		Outcome confirmation date	yyyy/mm/dd

Provide the status from the previous survey point to the survey point at the time of discontinuation/consent withdrawal.

*1: New-onset (asymptomatic) myocardial infarction is treated as an event, even without hospitalization.

*2: Includes old myocardial infarction, valvular disease, epicarditis, etc.

10.2 Central measurement inspection

When conducting routine blood sampling at the start of treatment, at 12 weeks, and at 96 weeks, collect blood for central measurement inspection and high-sensitivity CRP, folic acid, vitamin B12, NT-proBNP, serum iron, ferritin, and TIBC. An 18 mL sample of blood (approximately 6 mL of serum) is needed for this measurement. Sample collection and measurement will be conducted at SRL Medisearch Inc. (henceforth, "SRL"). The study physician records the inspection results from the inspection company in the case report form. Procedures, such as sample collection for central batch measurements, are specified separately in the SRL standard operating procedure.

Samples collected by the central batch measurement inspection will be stored according to the SRL standard operating procedure after measurements are completed, after which they are to be stored for 10 years. Samples should be discarded after the end of the storage period. Additionally, if items requiring inspection are added following the start of the study, then the stored samples will be used for measurement after obtaining the approval of the principal investigator-affiliated facility, the Foundation for the Promotion of Advanced Medicine, and the ethical review committee of the research consignor. The lead principal physician and study physician also obtain consent for sample storage and additional measurements with stored samples when consent is obtained from the patient for research participation.

10.3 Observation / inspection / reporting schedule

Observations, investigations, and inspections are conducted according to the following.

Item \ Time	Registration	Prior to start of treatment	Observation period											Simultaneous outcome	Discontinuation of participation / withdrawal of consent
			Start of treatment	2, 4, 6, 8, 10 weeks	12 weeks	16 weeks	24 weeks	36 weeks	48 weeks	60 weeks	72 weeks	84 weeks	96 weeks		
Eligibility determination															
Primary disease / complications / medical history		○													
PWV *1		○													
12-lead electrocardiogram / chest X-ray		●													
Height		○													
Weight			○	○	○	○	○	○	○	○	○	○	○		
Blood pressure			○		○		○		○		○		○		
Urinalysis: urine protein, urine Cr, PCR			○		○				○				○		
Blood test: HbA1c (only for diabetic patients) *2			○												
Blood test: Hb, MCV, Cr, eGFR, Alb, serum iron, ferritin, TIBC			○	○	○	○	○	○	○	○	○	○	○		
Blood test (central measurement): high-sensitivity CRP, folic acid, VB12, NT-proBNP, Fe, ferritin, TIBC			★		★								★		
Darbepoetin alfa administration status			→	→	→	→	→	→	→	→	→	→	→		

Iron formulation usage status			→	→	→	→	→	→	→	→	→	→	→		
Miscellaneous concomitant drug usage status			○	○	○	○	○	○	○	○	○	○	○		
Blood transfusion status			○	○	○	○	○	○	○	○	○	○	○		
Anemia influencing factor information			○	○	○	○	○	○	○	○	○	○	○		
Events / adverse events			→	→	→	→	→	→	→	→	→	→	→	→	→
Outcome information			→	→	→	→	→	→	→	→	→	→	→	→	→
Discontinuation / consent withdrawal															○
Inspection content			0	±1 week			±6 weeks								4 weeks
Case report form submission deadline	☆	☆			☆	☆	☆		☆		☆		☆	☆	
	After registration or within four weeks of end of final inspection of each case report form														

○ : Required

● : Implement before the start of treatment if there are no inspection results within 12 weeks prior to registration. Do not record in the case report form; report when an event occurs at the request of the event evaluation committee.

◇ : Implement only for Hb, Cr, eGFR (when inspection is implemented within eight weeks prior to registration, its latest value is allowed).

★ Central measurement: collect blood prior to darbepoetin alfa administration.

*1: Report the latest inspection values from 12 weeks prior to registration to five weeks after registration.

*2: Report the latest inspection values from eight weeks prior to treatment to start of treatment.

10.4 Definition of criteria related to observation / inspection / report items

10.4.1 Event

The following renal function deterioration or CVD events are set as events in this study. These definitions are provided separately.

Renal function deterioration	① Initiation of maintenance dialysis, ②kidney transplantation, ③50% decrease in eGFR, ④eGFR less than or equal to 6mL/min/1.73 m^2<br><br>＜Supplement＞<br>The eGFR decline rate is calculated with the average eGFR value from the start of treatment to up to 12 weeks as the baseline.<br><br>The first point where decreases are observed at three consecutive points is used for ③ 50% decrease in eGFR and ④ eGFR less than or equal to 6mL/min/1.73 m^2
CVD event	Fatal<br>① Death due to myocardial infarction, ② sudden death, ③ death due to congestive heart failure, ④ death due to arrhythmia, ⑤ cerebrovascular death, ⑥ death due to aortic dissection, ⑦ death due to ischemia of major organs other than the heart / brain, ⑧ other forms of cardiovascular disease
	Non-fatal<br>① Myocardial infarction*1, ② hospitalization due to angina pectoris, ③ hospitalization for ischemic heart disease requiring invasive intervention treatment, ④ hospitalization for congestive heart failure with chronic organic heart disease*2 (excluding arrhythmia), ⑤ hospitalization for severe arrhythmia and atrial fibrillation / atrial flutter, ⑥ hospitalization due to cerebrovascular disease, ⑦ hospitalization due to chronic arteriosclerosis obliterans, ⑧ hospitalization due to aortic dissection, ⑨ hospitalization due to ischemia of major organs other than the heart / brain<br><br>*1: New-onset (asymptomatic) myocardial infarction is treated as an event even without hospitalization.<br>*2: Includes old myocardial infarction, valvular disease, epicarditis, etc.

10.4.2 Factors affecting anemia

The following items are set as factors affecting anemia in this research.

Malignant tumors, hemorrhagic lesions, collagen disease, rheumatic disease, immunosuppressant or steroid use, other myelosuppressive factors

11. **Sample size and research period**

11.1 Sample size

The sample size is 2,000 patients.

11.2 Research period

The patient registration period and follow-up end date are as follows.

Patient registration period: approval date–March 31, 2016

Observation period end date: 96 weeks after the start of the last registered patient (simultaneous outcome survey)

12. Definition of endpoints

12.1 Primary endpoints

12.1.1 Renal function deterioration

Composite events of each event occurring 12 weeks after the start of treatment (see "10.4.1 Event – Renal function deterioration") are set as renal function deterioration. The number of days until occurrence is calculated, with 12 weeks after the start of treatment as the starting point.

12.1.2 CVD event

Composite events of each event occurring 12 weeks after the start of treatment are set as the CVD event. The number of days until occurrence is calculated, with 12 weeks after the start of treatment as the starting point.

12.2 Secondary endpoints

12.2.1 eGFR decline rate

For each patient, the slope of the linear regression of all eGFR from the start of administration to the final eGFR observation time point on the number of measured days from the start of administration is set as the eGFR decline rate.

12.2.2 Safety evaluation

The presence of adverse events and contents are evaluated for all adverse events occurring after the administration of darbepoetin alfa. MedDRA/J is used as a replacement dictionary for adverse events. The latest version at the time of analysis is used for the MedDRA/J. Those where the association with darbepoetin alfa is deemed "present" are defined as adverse drug reactions.

12.3 ERI

The following five indices are considered new candidates for the ESA response indices (ERIs) to be investigated.

- ERI-1A (index according to one time point at 12 weeks)

$$\frac{\text{DA administered dose 12 weeks after the start of treatment } (\mu\text{g})}{\text{Hb concentration 12 weeks after the start of treatment } (\text{g/dL}) \times \text{weight 12 weeks after the start of treatment } (\text{kg})}$$

- ERI-1B (index according to one time point at 12 weeks)

$$\frac{\text{DA administered dose 12 weeks after the start of treatment } (\mu\text{g})}{\text{Hb concentration 12 weeks after the start of treatment } (\text{g/dL})}$$

- ERI-2A (index that considers the cumulative administered dose up to 12 weeks and changes in Hb concentration)

$$\frac{\text{Total DA administered dose up to 12 weeks after the start of treatment (µg)}}{\text{Amount of change} \times \text{weight at 12 weeks (kg)}}$$

- ERI-2B (index that considers the cumulative administered dose up to 12 weeks and changes in Hb concentration)

$$\frac{\text{Total DA administered dose up to 12 weeks after the start of treatment (µg)}}{\text{Amount of change}}$$

- Change in Hb concentration (changes in Hb concentration up to 12 weeks)

$$\text{Hb concentration at 12 weeks (g/dL)} - \text{prior Hb concentration (g/dL)}$$

A similar index is created for the ERI index from the Hb concentration and darbepoetin alfa administered at 16 weeks and at 24 weeks in addition to that at 12 weeks, and the associations between these indices and event occurrence are also investigated. When the ERI index at 12 weeks favorably predicts event occurrence, then whether the ERI index composed of the values at week 10, 8, 6, 4, and 2 favorably predicted the event occurrence is confirmed in order.

New indices are further explored if no associations between these index candidates and renal function deterioration or CVD events were found.

13. Statistical analysis

13.1 Rationale for the sample size design

The objective of this research is to clarify the actual conditions of low ESA response cases in Japan, investigate factors related to low ESA response cases, and search for new ERIs. Therefore, in consideration of securing data for each type of exploratory analysis, a sample size of 2,000 was set as the scale of research where evaluations of low ESA response cases were thought to be possible.

The TREAT trial[8)] conducted internationally reported that renal events (end-stage renal failure or death) in the darbepoetin alfa group and the two-year occurrence rate of CVD events was approximately 23%–24%. If the event occurrence rate was set as 13.1 / 100 people/year from this result, then the number of events observed in two years with a sample size of 2,000 would be 480–568 patients at a 95% confidence interval, which is thought to be a sufficient number of events for the planned analyses.

13.2 Analysis set

The following groups are defined as the analysis sets. The acceptance or rejection of individual patients in each group is determined prior to the fixing of the data as needed. The main analysis set for efficacy evaluation is set as the "efficacy analysis set," but a similar efficacy analysis is implemented for "ACS."

13.2.1 Safety set

All patients who received darbepoetin alfa are included in the safety set.

13.2.2 Efficacy analysis set

The group excluding the following patients from the safety set is defined as the efficacy analysis set.

- Those who were ineligible after registration
- Those who discontinued darbepoetin alfa administration within 12 weeks after the start of treatment
- Those who do not have a single DA administration record 12 weeks after the start of treatment
- Hb concentration measurements are not obtained at the start of treatment and at 12 weeks

13.2.3 Administration Algorithm Compliance Set

The group that complied with the “Recommended darbepoetin alfa administration method” shown in Section 8.1.2, among the efficacy analysis set, is defined as the ACS.

13.3 Analysis items / methods

As a general rule, categorical data calculate frequencies and percentages, and continuous values are summarized with the summary statistical values of the number of patients, average value, standard deviation, minimum value, median value, and maximum value.

A significance level is not set because there are no hypotheses to be verified that are set in this research, but a two-sided P-value of 5% is set when using statistical test methods in an exploratory manner.

Details on analyses not recorded in this research protocol are described in the “Statistical Analysis Plan”

13.3.1 Renal function deterioration, CVD event investigation

A proportional hazards model that includes the following covariates is applied, and the factors that affect event occurrence are investigated. Covariates are added or decreased as required for the investigation of the model. Hb concentration values at each measurement time point up to 24 weeks instead of week 12 of administration are also investigated. The presence of diabetic complications, arteriosclerosis, and chronic inflammation should also be considered as stratification factors. These analyses are used to investigate the prediction equation for event occurrence from the covariates.

- Age; sex; weight; eGFR, serum albumin, hemoglobin concentration, high-sensitive CRP, NT-pro BNP, folic acid, vitamin B12, serum iron, serum ferritin, and TSAT 12 weeks after the start of treatment; the presence of diabetic complications, arteriosclerosis, chronic inflammation, malignant tumor complications, hemorrhagic lesion complications, collagen disease and rheumatic disease complications, concomitant immunosuppressant or steroid use, and other myelosuppressive factors; and the darbepoetin alfa administered dose (total administered dose, administered dose 12 weeks after the start of treatment).

Next, the cut-off values of each ERI are gradually increased from the minimum value to the maximum

value; a time-dependent ROC curve of the survival time data with censoring is created, and the AUC is compared. If the cut-off value is set from the ROC curves of each obtained ERI, then these are divided into two groups at the cut-off value, Kaplan-Meier plots are created, and the annual event occurrence rate (human-year method) and 95% confidence intervals are estimated. The cut-off values of the ERIs may also be investigated using the cross-validation method, if considered useful.

13.3.2 eGFR decline rate

A linear mixed model, including the covariates shown in the previous sections, is applied to all the eGFR values from the start of administration to the final eGFR observation time point in order to search for factors that affect eGFR decline.

13.3.3 Safety evaluation

The number of occurrences is tabulated for all adverse events and adverse drug reactions. The tabulation of frequency according to content is performed according to the system organ class (SOC) and preferred term (PT) of MedDRA/J.

13.4 Intermediate analysis / intermediate tabulation

No intermediate analyses are conducted in this research.

Intermediate tabulations for regular reports (e.g., patient background, patient registration status, and case report collection status) are conducted in this research.

14. **Input and submission of case report form**

14.1 Form and submission deadline

The case report form of this research and its submission deadlines are as follows.

Case report form title	Submission deadline
At the time of registration	Within four weeks after registration, up until the start of treatment
Prior to the start of treatment	Within four weeks after the end of inspections at the start of treatment
At the start of treatment<br>Two weeks after the start of treatment<br>Four weeks after the start of treatment<br>Six weeks after the start of treatment<br>Eight weeks after the start of treatment<br>10 weeks after the start of treatment<br>12 weeks after the start of treatment	Within four weeks after the end of inspections 12 weeks after the start of treatment
16 weeks after the start of treatment	Within four weeks after the end of inspections 16 weeks after the start of treatment

24 weeks after the start of treatment	Within four weeks after the end of inspections 24 weeks after the start of treatment
36 weeks after the start of treatment<br>48 weeks after the start of treatment	Within four weeks after the end of inspections 48 weeks after the start of treatment
60 weeks after the start of treatment<br>72 weeks after the start of treatment	Within four weeks after the end of inspections 72 weeks after the start of treatment
84 weeks after the start of treatment<br>96 weeks after the start of treatment	Within four weeks after the end of inspections 96 weeks after the start of treatment
Darbepoetin alfa administration status<br>Iron formulation details<br>Blood transfusion details	Submitted according to the submission time of each period
Fatal event<br>Non-fatal event<br>Renal function deterioration event<br>Adverse event	Submitted according to the submission time of each period<br>(final outcome is within four weeks after the end of simultaneous outcome survey)
Discontinuation of research participation	Within four weeks after the confirmation of discontinuation / consent withdrawal (only for applicable patients)

14.2 Input method

The following items should be observed when inputting information.

・ A case report form input should be conducted according to the "online input system operation manual".

・Inputting and corrections will be conducted by the principal physician or physician. However, the input and correction of sections that do not involve medical decisions (e.g., posting from medical records) can be conducted by research collaborators under the supervision of the lead principal physician or study physician.

・Refer to "Appendix 1 Anonymization number comparison table" when inputting information, and confirm that the medical record belongs to the applicable target patient.

・After completing the case report form input, print this and store it in the medical records.

Contact the data center for questions regarding the input method.

14.3 Case report content confirmation and questions

1) The data center will confirm the following items for the case report from that signed online.

・Incomplete input

・Protocol consistency

・Case report form content consistency

2) The data center will input the points of inquiry into the question list on the website.

3) The lead principal physician or study physician will confirm the question list and make inputs or corrections on the website or enter comments in the answer column of the question list.

15. Research management

15.1 Progress management

The data center will create a progress report of this research, which summarizes the patient registration and case report form collection, and report to the principal investigator and statistical analysis manager.

15.2 Research monitoring

Protocol compliance in this research is confirmed by central monitoring based on records, such as patient registration forms and case report forms collected in the data center.

16. Various committees

16.1 Steering committee

This committee will, together with the principal investigator, comprehensively manage and operate the implementation of this research, create the research protocol and explanatory consent document, and conduct revisions during research implementation.

16.2 Independent data monitoring committee

The purpose of this committee is to conduct objective evaluations regarding the progress and safety of this research and to propose changes or discontinuation of the research to the principal investigator. Detailed operations and procedures are specified separately in the “Independent data monitoring committee procedure manual.”

16.3 Event evaluation committee

This committee will conduct medical decisions on events occurring during research from an independent perspective at the request of the principal investigator. Detailed operations and procedures are specified separately in the “Event evaluation committee procedure manual.”

17. Ethical items

17.1 Rules to be observed

This research will be conducted in accordance with the spirit of the “World Declaration of Helsinki,” Ethical Guidelines for Clinical Research (fully revised on July 31, 2008; Ministry of Health, Labor, and Welfare), and the research protocol.

17.2 Explanatory document / consent form (format) creation and amendment

The lead principal physician will create an explanatory document/consent form (format), which will be submitted to the ethics review board prior to the start of the research, where their approval is to be obtained.

17.2.1 Items to be described in explanatory document

The explanatory document should include the following items stipulated in the “ethical guidelines for clinical research. However, there should be no descriptions that intentionally direct the patient.

1) Fact that participation in this clinical research is voluntary.
2) Fact that the patient will not experience any disadvantages as a result of not consenting to participate in this clinical research.
3) Fact that subject patients or their representatives can withdraw the informed consent they provided themselves at any time without any disadvantages.
4) Reason for selection as a target patient.
5) Significance, purpose, method, and duration of this clinical research.
6) Name and job titles of researchers.
7) Expected results of this clinical research, advantages expected from the participation of this clinical research, possible risks, any inevitable physical or psychological discomfort, and treatment after completion of this clinical research.
8) Fact that patients can obtain or browse materials related to the clinical research plan and clinical research methods upon request by the patient or their representative, within a scope that does not hinder the protection of personal information of other target patients and the novelty of this clinical research.
9) The results of this clinical research could be provided to other institutions after the ethics review board show that the handling of personal information, name of the institution to which this information is provided, and the purpose of use at the recipient institution is valid.
10) It is possible that patent rights, etc., will be created as a result of this clinical research, and the attribution of those rights are created.
11) The results of this clinical research may be published after taking measures to ensure that the patients cannot be identified.
12) Funding sources for this clinical research, possible conflicts of interest, and relationships with related organizations.
13) Storage and usage methods of samples, and storage duration.
14) Information for questions regarding this clinical research as well as contacts for complaints
15) Necessary measures, such as insurance to compensate for health damage caused to target patients due to this clinical research

17.2.2 Items to be stated in consent form

The following items should be stated in the consent form.

1) Clinical research name
2) Explanatory document creation date, version
3) Explanation date, name, and seal or signature line of lead principal physician or study physician
4) Consent date, name and seal or signature line of patient
5) If a representative is present, then consent date, relationship, name, and seal or signature line of representative
6) The statement that the explanation content is understood, and that consent to participate in research is given.
7) Implementing medical institution name

17.2.3 Items to be stated in consent withdrawal form

The following items should be stated in the consent withdrawal form.

1) Clinical research name
2) Name and seal or signature line of lead principal physician or study physician
3) Consent withdrawal date, name and seal or signature of subject patient
4) If a representative is present, then consent withdrawal date, relationship, and name and seal or signature of representative
5) Statement that consent to participate in research is withdrawn
6) Implementing medical institution name

If the lead principal physician obtained new information relating to patient consent after the start of research or determined that amendments were needed in the explanatory document/consent form, then these were amended. Examples of new findings relating to patient consent refer to information on new adverse events associated with the applicable treatment method or information associated with the development of new treatments for the applicable disease. If it is determined that the amendment contents would affect the decision of the patient to continue in the research, the amended explanatory document/consent form is to be submitted to the ethics review board of the affiliated medical institution, and after their approval is obtained, this will be explained again to the patient and their consent will be obtained.

17.3 Obtaining implementation approval at implementing medical institution

17.3.1 Inspection of whether research can be conducted

The lead principal physician will submit an application to the head of the medical institution to which they belong prior to the implementation of this research and acquire approval from the ethics review board and implementation approval by the head of the medical institution. If an ethics review board

cannot be established at the applicable medical institution, then the ethics review board may be set up jointly by the heads of multiple medical institutions or heads of other medical institutions.

17.3.2 Continuation inspection

The lead principal physician will provide an overview of the current status of this research to the head of the research implementing medical institution at the request of the ethics review board of each research implementing medical institution in order to obtain continuation inspections of the ethics review board.

17.4 Patient disadvantages and advantages

17.4.1 Disadvantages

This research is a survey on the actual conditions of treatment, so no treatment interventions are conducted. However, blood tests (central measurements) (at start, after 12 weeks, after 96 weeks) are needed as a result of participating in this research.

17.4.2 Advantages

It is expected that useful information on treatments that would affect the prognosis of CKD patients will be obtained from the information in this research, and it is thought that this will lead to widespread social advantages for CKD patients.

17.5 Protection of personal information

Every effort will be made by all people involved in this research to protect personal information as data handlers. Linkable anonymization will be adopted based on the definition of 1-3(8) and (9) of the “Ethical Guidelines for Clinical Research” as the handling method for the applicable research data.

A format is used in which information, such as the name of each patient or their medical records, are anonymized by assigning a patient registration number issued by the data center, and personal information (e.g., names) is not inferred when submitting data to outside the applicable facility. The “Appendix 1 Anonymization Number Comparison Table,” which is the linked table with personal information, is strictly managed at each facility and is used only when inquiring research data (linkable anonymization).

18. **Research costs and burdens**

18.1 Funding sources and financial relationships

Kyowa Hakko Kirin Co., Ltd is providing funding for this research. The funder, Kyowa Hakko Kirin Co., Ltd., proposes and provides information on the concepts of this research, but they are not involved in the analysis and interpretation of the results.

18.2 Conflicts of interest

This research is funded by Kyowa Hakko Kirin Co., Ltd., a company that manufactures and sells the drug darbepoetin alfa used in this research. However, this research is implemented mainly by the

principal investigator, and the research operation and DM are contracted to the Translational Research Informatics Center (TRI), which is a third-party institution that is independent from the affiliated facilities of the principal investigator. Therefore, this company would not be able to have any effect on the analysis or interpretation of the results of this research, and this research is implemented by a system that ensures impartiality. Inspections on conflicts of interest associated with participation in this research are conducted according to what was agreed upon at each institution to which the researchers involved in this research belong.

18.3 Medical expenses during research participation

The medical care received by patients participating in this research is implemented in the same manner as normal treatment, and the expenses are covered by the patient's health insurance and out-of-pocket expenses. Up to 3,000 yen in burden reduction fees are given at the time of blood collection for central measurements in order to reduce the burden on patients participating in this research. The payment method is conducted according to what was agreed upon at each facility.

18.4 Compensation for health hazards

This research uses drugs that are already commercially available, so patients who experience serious side effects as a result of doses specified in this research will be handled in the same way as in normal medical care. These are also subject to relief benefit applications under the relief system for sufferers from adverse drug reactions. The lead principal physician, etc., will be enrolled in liability insurance in case a liability occurs.

19. **Amendment of Research Protocol**

The principal investigator will make efforts to collect the associated internal and external research information, even during the research period. Cases where amendments to the research protocol are needed will involve the creation of an amended research protocol and its amendment history (amendment content and its reason) after discussing with the steering committee. When implementing amendments, report to the head of the affiliated medical institution, and, if necessary, obtain inspection and approval from the ethics review board.

When the principal investigator reports on the amendment of the research protocol, the lead principal physician will carry out the amendment procedure according to the regulations of each implementing medical institution.

The principal investigator will contact the lead principal physician, study physician, committee, and data center if patient registration needs to be suspended during the amendment.

Cases where amendments of the explanatory document are needed according to the amendment content of the research protocol will be handled similarly to that of the research protocol.

20. Research completion and early discontinuation

20.1 Completion of research

The time point at which the data are fixed by the data center is set as the completion of the research. The principal investigator will report the completion of the research to the lead principal physician, study physician, each committee, data center, and the statistical analysis manager, and the lead principal physician who received the report will report this to the head of the affiliated medical institution and the associated departments within the medical institution.

20.2 Early discontinuation / suspension of research

20.2.1 Rules for early discontinuation / suspension of research

Early discontinuation or suspension of the research will be implemented in the following patients.

1) When it is determined as a result of evaluating progress reports, the principal investigator would find it difficult to complete the research due to delays in patient registration.
2) When it is determined as a result of investigating serious adverse event reports that there are safety problems regarding the implementation of this research.
3) When it is determined as a result of evaluating associated information obtained from outside this research (e.g., research articles, conference presentations) that there are safety problems regarding the implementation of this research or if there is no longer any significance in continuing this research.

20.2.2 Early research discontinuation decision procedure

If an event that falls under the early discontinuation criteria occurs, the principal investigator must host an independent data monitoring committee and receive recommendations. The principal investigator will consider their recommendations and determine the need for early discontinuation upon discussion with the steering committee. If choosing not to follow the recommendations, the principal investigator must report the reason for doing so with an independent data monitoring committee.

If this research is discontinued or interrupted, the principal investigator will promptly report this and the reason for doing so to the lead principal physician, study physician, committee, advisors, and data center. The lead principal physician will promptly report this to the subject patients and take appropriate measures. Additionally, the lead principal physician will report this to the head of the research implementing medical institution and the associated departments within the medical institution, following the procedures established by the applicable medical institution. The head of the research implementing medical institution will report this and the reason for discontinuation/suspension to the ethics review board.

21. Record keeping

The principal investigator, as well as the lead principal physician or study physician, will store records relating to the implementation of this research (documents and electronic records) for 10 years after the

completion of this research. The data center promptly will transfer the research-related documents it is storing to the principal investigator after submitting the statistical analysis report.

22. Publication of research and attribution of results

22.1 Clinical research registration

This research will be registered in the National University Council of Japan-established Clinical Trials Database (UMIN) and ClinicalTrials.gov database prior to the start of patient registration based on the recommendations of the International Committee of Medical Journal Editors (ICMJE).

22.2 Attribution of results

22.2.1 Presentation of research results

The articles and conference presentations of the results obtained from this research will be determined after a suitable discussion with the steering committee, which includes the principal investigator. Individuals presenting research results will obtain inspections and approval from the steering committee, which includes the principal investigator.

22.2.2 Attribution of data

The data collected from this research is attributed to the principal investigator.

23. **Research organization**

23.1 Principal investigator

Seiichi Matsuo Department of Nephrology, Nagoya University Graduate School of Medicine

23.2 Co-principal investigator

Masaomi Nangaku Department of Nephrology and Endocrinology, University of Tokyo Graduate School of Medicine

23.3 BRIGHTEN Research Secretariat

Department of Nephrology and Endocrinology, University of Tokyo Graduate School of Medicine

Coordinator: Masaomi Nangaku, Takehiko Wada

23.4 BRIGHTEN TRI Secretariat

Translational Research Informatics Center

Coordinator: Mitsuru Maeda

23.5 Steering committee

Seiichi Matsuo Department of Nephrology, Nagoya University Graduate School of Medicine

Masaomi Nangaku Department of Nephrology and Endocrinology, University of Tokyo Graduate School of Medicine

Hideki Hirakata Division of Nephrology and Dialysis Center, Japanese Red Cross Fukuoka Hospital

Ichiei Narita Division of Clinical Nephrology and Rheumatology, Niigata University Graduate School of Medical and Dental Sciences (Department of Medicine II)

Takashi Wada Department of Laboratory Medicine, Institute of Medical, Pharmaceutical and Health Sciences, Faculty of Medicine, Kanazawa University

Terumasa Hayashi Department of Kidney Disease and Hypertension, Osaka General Medical Center

Shoichi Maruyama Department of Nephrology, Nagoya University Graduate School of Medicine

23.6 Statistical analysis manager

Tatsuo Kagimura Translational Research Informatics Center

23.7 Event evaluation committee

Chairman Hiroyasu Yamamoto Internal Medicine, Atsugi City Hospital

Chairman Hiroki Hase Department of Nephrology, Toho University Ohashi Medical Center

Chairman Masahiro Kamouchi Department of Health Care Administration & Management, Kyushu University Graduate School of Medical Science

23.8 Independent data monitoring committee

Chairman Enyu Imai Nakayamadera Imai Clinic

Chair Kyoichi Mizuno Mitsukoshi Health and Welfare Foundation

Chair Manabu Iwasaki Department of Computer and Information Science, Faculty of Science and Technology, Seikei University

23.9 Study Group Advisors

Tadao Akizawa Department of Nephrology, Showa University School of Medicine

Yoshiharu Tsubakihara Department of Comprehensive Kidney Disease Research, Osaka University Graduate School of Medicine

23.9.1 Data center

TRI Data Center

Coordinator: Emiko Uno, Hideki Kawano

23.9.2 Blood test (central measurement) inspection institutions and sample collection / transportation

SRL Medisearch Inc.

President and CEO Hiroyuki Sekiguchi

23.9.3 Implementation of business concluded in consigned research contract with medical institutions

MC&P Co., Ltd.

President and CEO Seiichi Uezono

23.9.4 Research consignor

Kyowa Hakko Kirin Co., Ltd.

President and CEO Nobuo Hanai

23.9.5 Anticipated implementing medical institution and lead principal physician

See attachment.

24. References

1) Lozano R, Naghavi M, Foreman K, et al. Global and regional mortality from 235 causes of death for 20 age groups in 1990 and 2010: a systematic analysis for the Global Burden of Disease Study 2010. *Lancet*. 2012;380(9859):2095-2128.
2) Ministry of Health, Labor and Welfare (2011) overview of vital statistics (fixed number). Table 6: Gender-based number of deaths, mortality rate, and composition percentage ranked by cause of death (up to 10th place) (per 100,000 people). 2012 http://www.mhlw.go.jp/toukei/saikin/hw/jinkou/kakutei11/dl/10_h6.pdf
3) Japanese Society of Nephrology "GFR estimation equation project for Japanese people"
4) CKD Clinical Guideline 2012
5) 2008 edition JSDT Guidelines for the treatment of renal anemia in patients with chronic kidney disease
6) 2012 edition KDIGO Clinical Guideline
7) Singh AK, Szczech L, Tang KL, et al. Correction of anemia with epoetin alfa in chronic kidney disease. *N Engl J Med*. 2006;355(20):2085-2098.
8) Pfeffer MA, Burdmann EA, Chen CY, et al. A trial of darbepoetin alfa in type 2 diabetes and chronic kidney disease. *N Engl J Med*. 2009, 361(21):2019-2032.
9) Szczech LA, Barnhart HX, Inrig JK, et al. Secondary analysis of the CHOIR trial epoetin-alpha dose and achieved hemoglobin outcomes. *Kidney Int*. 2008;74(6):791-798.
10) Solomon SD, Uno H, Lewis EF, et al. Trial to Reduce Cardiovascular Events with Aranesp Therapy (TREAT) investigators: erythropoietic response and outcomes in kidney disease and type 2 diabetes. *N Engl J Med*. 2010;363(12):1146-1155.
11) Imai E, Matsuo S, Makino H, et al. Chronic Kidney Disease Japan Cohort study: baseline characteristics and factors associated with causative diseases and renal function. *Clin Exp Nephrol*. 2010;14(6):558-570.

Appendix 1 Anonymization number comparison table

No	Patient name	Gender	Date of birth	Medical record number	Patient identification code*	Registration number	Registration date	Registering study physician
1		Male / female	year month day			—	year month day	
2		Male / female	year month day			—	year month day	
3		Male / female	year month day			—	year month day	
4		Male / female	year month day			—	year month day	
5		Male / female	year month day			—	year month day	
6		Male / female	year month day			—	year month day	
7		Male / female	year month day			—	year month day	
8		Male / female	year month day			—	year month day	
9		Male / female	year month day			—	year month day	
10		Male / female	year month day			—	year month day	
11		Male / female	year month day			—	year month day	
12		Male / female	year month day			—	year month day	
13		Male / female	year month day			—	year month day	
14		Male / female	year month day			—	year month day	
15		Male / female	year month day			—	year month day	
16		Male / female	year month day			—	year month day	
17		Male / female	year month day			—	year month day	
18		Male / female	year month day			—	year month day	

* Do not use personally identifiable items, such as medical record numbers and names. Note): The lead principal physician of each medical institution or a representative will store this information in a responsible manner.

How to use anonymization number comparison table

No personally identifiable information can be included in the case report form in clinical research to ensure privacy.

All information is managed by registration number.

Therefore, the medical institution will need to manage the correspondence between the individual patient and registration number.

The patient identification code is the only information that can be used to identify patients without disclosing personal information.

However, the case report form must be accurately created without making any mistakes with the patient.
Therefore, a patient identification code must be uniquely assigned and managed.

This is personally identifiable information, so this cannot be disclosed outside the facility.

＜Example＞

No	Patient name	Gender	Date of birth	Medical record number	Patient identification code[*1]	Registration number	Registration date	Registering study physician
1	Taro Yamada	Male / female	March 4, 1937	Y200512345	○○○-001	001-001	January 25, 2006	Taro Kobe
2	Hanako Suzuki	Male / female	April 5, 1948	S199998765	○○○-002	001-004	March 3, 2006	Taro Kobe

Managed at facility

Managed at the data center

Please assign an arbitrary code that does not include personal information and is unique to the patient identification code.

＜Bad examples＞

yamataro ·································· Can be guessed that the name is “Taro Yama○”

YT19370304······························ Can be guessed that the individual has the initials “YT” and was born March 4, 1937

Y200512345 ······························ Cannot be used because the medical record number easily identifies the individual

0783039117······························ Personal information such as the patient’s phone number cannot be used

<u>Ensure that this anonymization number comparison table is used to confirm the correspondence between the patient medical records and registration numbers when creating the case report form.</u>

This table can be created for each researcher or managed by each medical institution.

Appendix 2 Reporting and response manual when serious adverse event occurs

Report / response procedure

① Initial report

【Report to related parties】

When an event or information defined by "9.4.1 Adverse events that much be reported" has occurred, the study physician of the applicable patient will promptly report to the lead principal physician and head of the affiliated research institution upon becoming aware of the disturbance. The report to the principal investigator is automatically reported by inputting this in "Adverse events" in the case report form.

【Review by an independent data-monitoring committee】

The principal investigator will promptly contact the chair of the independent data monitoring committee as needed, determine the need for emergency measures (including registration suspension), and determine the policy. If emergency measures are needed, then the emergency measures and their reasons are reported to the research participating facilities and data center.

Additionally, if the reported content is an event that affects the continuation of this research, the principal investigator will implement appropriate measures, such as temporary suspension of research, without awaiting the decision from the head of the medical institution.

【Reporting / response to participating facilities】

When the principal investigator has determined that the applicable event is a "serious adverse events associated with the research" in the guidelines, this event is reported to the principal physician of each facility.

The principal physician who received this report will follow the procedure of each facility and report to the head of the medical institution, etc.

【Reporting to the Minister of Health, Labor and Welfare】

The head of the medical institution to which the principal investigator belongs will promptly report to the Minister of Health, Labor and Welfare using Attachment 2 when the serious adverse event that occurred corresponds to an "unexpected serious adverse event".

② Additional report

When additional information arises or when detailed information is requested from the principal investigator, the lead principal physician will report the requested information to the head of the medical institution and principal investigator.

*Supplement

Voluntary reporting of individuals who handle pharmaceutical products or medical devices based on Article 77-4-2-2 of the Pharmaceutical Act (Act No. 145 of 1960) is the responsibility of the study physician or lead principal physician and is done in accordance with the regulations of the applicable medical institution.
