# Supplementary material for: Hyporesponsiveness to erythropoiesis-stimulating agent in non-dialysis-dependent CKD patients: The BRIGHTEN study
Source: PLoS One. 2022 Nov 29;17(11):e0277921. doi: 10.1371/journal.pone.0277921 (PMC9707758; doi:10.1371/journal.pone.0277921)
Supplement: Attachment — Submitted filename: ResponsletterR2.docx [file pone.0277921.s006.docx]

**RESPONSES TO THE COMMENTS**

The original comments of the reviewers and our responses to the comments are as follows:

**Responses to Reviewer # 1’s Comments:**

#1: The authors were somewhat responsive to my previous comments. I still don't understand why the manuscript has been submitted as a "Clinical Trial", versus a standard Research Paper. Please clarify further.

Response: We appreciate for your comments and agree with your criticism. We would like to change the category.
